# Burnout in residents during the first wave of the COVID-19 pandemic: a systematic review and meta-analysis

**DOI:** 10.3389/fpsyt.2023.1286101

**Published:** 2024-01-24

**Authors:** Ricard Navinés, Victoria Olive, Diego Hidalgo-Mazzei, Klaus Langohr, Eduard Vieta, Rocio Martin-Santos

**Affiliations:** ^1^Department of Psychiatry and Psychology, Hospital Clinic, Institut d Investigacions Biomèdiques August Pi I Sunyer (IDIBAPS), Centro de Investigación Biomédica en RED en Salud Mental (CIBERSAM), Barcelona, Spain; ^2^Functional Unit of Psychiatry, Department of Medicine, Neuroscience Institute, University of Barcelona (UB), Barcelona, Spain; ^3^Department of Occupational Risk and Prevention, Hospital Clinic, University of Barcelona (UB), Barcelona, Spain; ^4^Department of Statistics and Operations Research, Universitat Politècnica de Catalunya, Barcelona Tech, Barcelona, Spain

**Keywords:** systematic review, meta-analysis, burnout, residents, COVID-19, risk factors

## Abstract

**Introduction:**

The high prevalence of burnout in resident physicians is expected to have increased as a result of the expansion of the pandemic. We conducted a systematic review with a meta-analysis of studies conducted during the first wave of the COVID-19 pandemic on burnout in residents and potential associated risk factors.

**Methods:**

The search was done in the Web of Science, MEDLINE, Scopus, and Lillac databases (April 2020–October 2021) using *a priori* protocol based on the PRISMA guidelines. The Newcastle Ottawa Scale was used to assess the risk of bias in the included studies. We estimated the pooled prevalence (95% CI) of burnout and the prevalence ratio (95% CI) of each risk factor associated.

**Results:**

We included 23 studies from 451 potential initial articles and those written in the English language; all of the collected studies were cross-sectional with anonymous online surveys, involving 4,998 responders (34%), of which 53.2% were female responders, 51% were R1-2, and 71% were in direct contact with COVID-19 patients. Eighty-seven percent presented a low-to-moderate risk of bias. Publication bias was not shown. The estimated pooled prevalence of burnout was 40% (95% CI = 0.26 – 0.57). Burnout was associated with psychiatry history (PR = 4.60, 95% CI = 1.06 – 20.06). There were no differences by gender, civil status, children in-charge, year of residency, or time exposure to COVID-19.

**Discussion:**

The overall prevalence of burnout in residents during the first wave of the pandemic was in line with the results described in this collective before the pandemic. The presence of a psychiatry history was a potential burnout risk factor, suggesting a high vulnerability during the peak of the stress period and the need to implement mental health surveillance for this subgroup.

## 1 Introduction

The COVID-19 pandemic has made a significant impact on the mental health of workers, especially those who work on the frontline or who have been exposed to extreme and continuous pressure (1–5).

Before the pandemic, health professions were already considered “highly stressful” in themselves and, therefore, had frequent professional leave (6). Research on stress in resident physicians shows that this group of professionals is especially vulnerable (7). Chronic uncontrollable work stress is associated with a minor motivation, insight, and empathy, with a loss of concentration, impaired cognitive skills, and detachment from work, which are the characteristics of a worker with burnout (8, 9). The WHO, in the new version of the international diagnostic classification (10), includes burnout as an “occupational phenomenon” and incorporates in its description, in addition to feelings of exhaustion, greater mental distancing toward work, feelings of negativism or cynicism related to work, and decreased professional effectiveness^1^.

The “burnout syndrome” has been assessed clinically by the Maslach Burnout Inventory (MBI) (11, 12), which measures its three dimensions: emotional exhaustion (EE), depersonalization (DP), and personal accomplishment (PA). EE refers to the experience of being emotionally exhausted by the demands of work. DP assesses the degree to which each one recognizes attitudes of coldness and distance from people. Finally, PA measures feelings of self-efficacy and personal accomplishment at work. Systematic reviews with meta-analysis carried out on residents in the previous years of the COVID-19 pandemic, using the MBI as a burnout tool, found a high prevalence of burnout syndrome in residents, ranging from 35 to 51% (13, 14). National longitudinal studies of burnout in a similar period and using the MBI tool for burnout assessment showed that female gender, few sleep hours, surgery specialties, work overload, and young and older residents were risk factors of burnout in residents (15–19). Meanwhile, empathy and quality of life were protective factors in the period of training (15, 18).

The prevalence of burnout in resident physicians as a result of the expansion of the pandemic, being already high in this group, is expected to have increased, with potential negative effects on their physical and mental health. In this context, the objective of this study was to systematically review the web-based surveys published since the declaration of the pandemic in March to the end of the first wave in July 2020 on the prevalence of burnout syndrome and its associated potential risk factors.

## 2 Methods

### 2.1 Registration and reporting

We performed a systematic review of the literature to identify articles discussing the prevalence of burnout in residents and the potential risk factors associated with the pandemic. The Preferred Reporting Items for Systematic Reviews and Meta-Analyses (20) consensus was followed in the completion of this systematic review and meta-analyses and elaboration in advance of the protocol study (see Supplementary material). We electronically searched the literature in more than four databases (Web of Science, Scopus, Lilacs, and PubMed) with MeSH and keywords with subject headings “resident burnout” OR “trainee burnout” AND “COVID-19” for entries published from database inception through March 2020 to October 2021. Potential articles were reviewed first by title and abstract only, next by full text, and finally by analyzing eligible studies in detail by two reviewers. References of the included articles were reviewed to identify additional citations.

The inclusion criteria were as follows: (1) confirmed burnout syndrome in residents during the COVID-19 pandemic using a validated tool (i.e., MBI) (11, 12); (2) cross-sectional studies with and without comparator and before- and after-pandemic studies; (3) studies conducted during the first wave of the pandemic; and (4) studies published in English or Spanish in a peer-reviewed journal. The exclusion criteria were as follows: (1) other physicians apart from residents in training; (2) studies that assessed burnout exclusively outside the pandemic period; (3) editorials, reviews, case reports, commentaries, experimental, interventional, and qualitative studies; and (4) studies with a sample size of N<50 participants.

The main outcome was the prevalence of burnout syndrome during the COVID-19 pandemic or burnout dimensions. Additional outcomes were the factors associated with burnout prevalence in residents during the first wave of the pandemic.

We used the Newcastle Ottawa Quality Assessment Scale for observational studies to assess the quality and risk bias of eligible articles, which includes nine items related to selection, comparison, and outcomes (21). For each item, a start is awarded, except for comparison and clear variables that can receive up to two starts. The studies with more than six starts (maximum 8) were classified as having a low risk of bias, studies with 5–6 starts as having a moderate risk of bias, while studies with < 5 starts were considered as having a high risk of bias. Two reviewers rated each study, assessing a score out of eight possible points. Discrepancies were resolved by consensus.

Data were extracted independently by two authors including authors' names, date of publication, country, study type, sample size, type of specialty, gender, mean (SD) age and range, civil status, children in-charge, year of residence, direct contact with COVID-19 patients, burnout tool, burnout prevalence of syndrome and/or dimensions, and risk/protector factors associated with burnout [sociodemographics, history of mental disorders (i.e., depression/anxiety), frequency or a number of COVID-19 patients attended, positive COVID-19 one-self or colleague, having adequate access to personal protective equipment (PPE), changes per rotation/vacation, or increase/decrease of weekly work hours]. Discrepancies were resolved by consensus with a third MD researcher.

### 2.2 Strategy analysis

First, we did a systematic synthesis of the findings from the included studies around burnout outcomes and risk factors. Second, a quantitative synthesis was used if the included studies were sufficiently homogeneous. We performed meta-analyses using, as a primary effect size, the prevalence of burnout and dimensions, and, as secondary effect sizes, the prevalence ratios associated with burnout prevalence during the pandemic. Statistical heterogeneity among studies was inspected through the *I*^2^ index (low heterogeneity ≤ 25%, moderate 50%, and high >75%) and Cochrane's *Q* statistic (*p* < 1) and is reported for all analyses. Independent of the corresponding χ^2^-test for homogeneity, for the sake of coherence, the random-effects models were employed for the estimation of both burnout prevalence and prevalence ratios. Furthermore, in the case of the estimation of the prevalence ratios, the weights given to each study, i.e., the proportion of the total variability in the effect size estimates using random-effect models, are provided in the forest plots.

Subgroup meta-analysis estimates were pooled based on population characteristics such as gender, civil status, children in-charge, year of residency and specialization; burnout tool; and different settings (direct or no direct contact with COVID-19 patients) if we found data to carry out the meta-analysis. A prevalence ratio (PR) of 1 means that the prevalence of the event, in this case, burnout, is identical in the exposed and control or reference group, whereas a PR greater (less) than 1 indicates that the prevalence of burnout is higher (lower) in the exposed group. The statistical significance at a significance level of 0.05 of the estimated PR can be inferred from the 95% CI. If the CI includes the value 1, the estimated PR is not statistically significantly (*p* > 0.05) different from 1. A graphical exploration of a potential publication bias by means of a funnel plot was carried out if, at least, 10 or more studies were included in the analysis.

All analyses were performed with the statistical software package R (The R Foundation for Statistical Computing), version 4.1.1; in particular, we used the contributed R meta package (22).

## 3 Results

### 3.1 Search results

**Figure 1:**
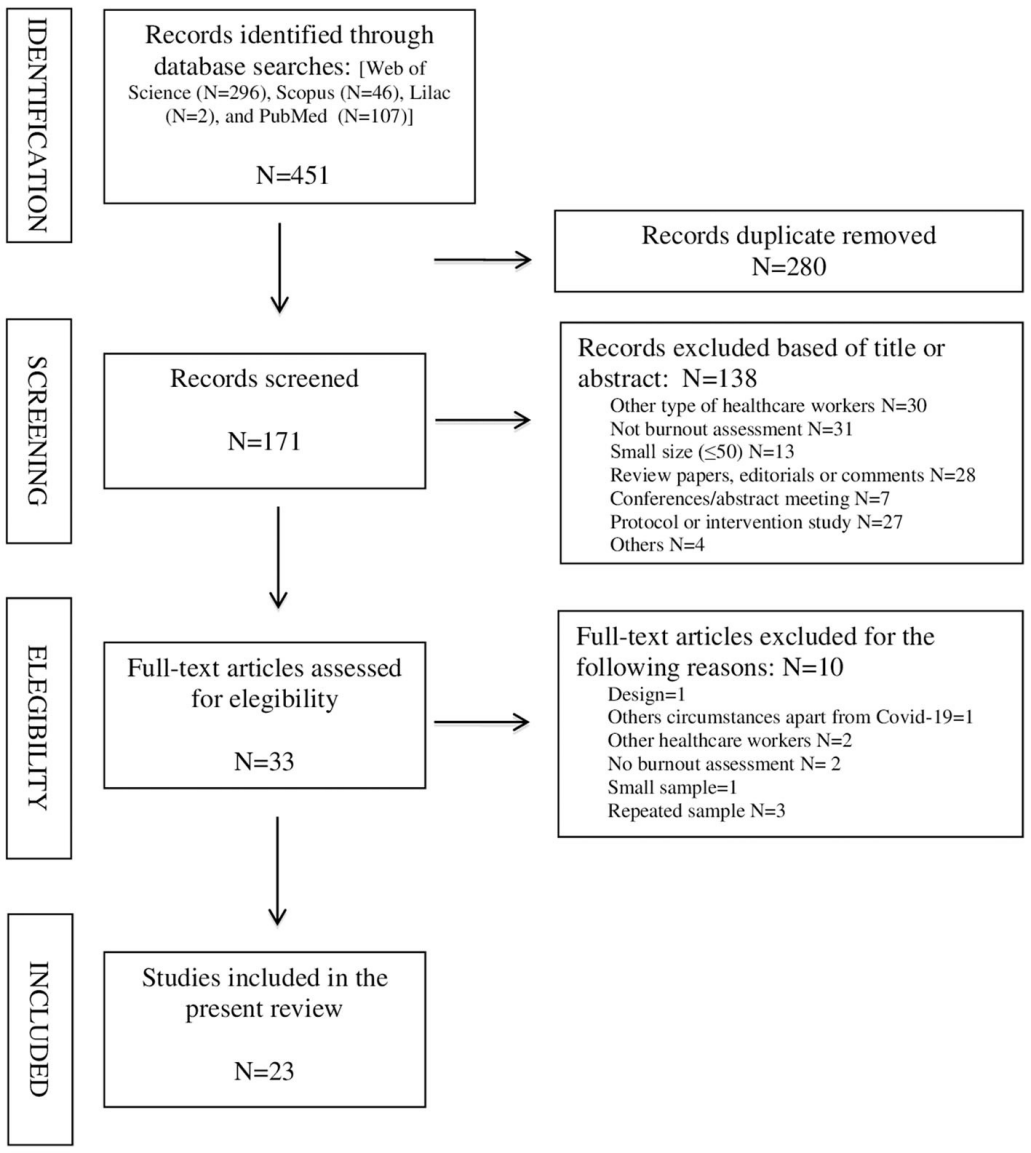
Flowchart of considered and finally selected articles according to the PRISMA statement.

The preliminary research of electronic databases yielded 451 potential articles. After removing 280 duplicated records, 138 articles were excluded based on the review of titles and abstracts, and 33 were retrieved for full-text evaluation. After the application of the exclusion criteria, 23 articles met the criteria for final inclusion. The flowchart of the systematic review is shown in Figure 1.

### 3.2 Characteristics of included studies

The characteristics of the 23 studies included in the systematic review and meta-analysis are summarized in Table 1 (23–45). All studies were cross-sectional with an anonymous online survey. In total, eight of 23 (34.8%) studies were conducted in North America (23–29, 45), 6 (26.1%) in Europe (30–35), 5 (21.7%) in Asia (36, 37, 39–41), 2 (8.7%) in South America (42, 43), and 1 in Africa (4.4%) (38), but 1 (4.4%) was an international study with countries from different continents (44). Twelve studies (52.2%) were national studies (23, 25, 27–31, 34–37, 39). The response rate ranged from 94.8 to 7.9%. Ten studies included residents from all specialties (30.3%) (26, 32, 37–40, 42–45), and eight were doing surgery (34.8%) (23, 25, 27–29, 34, 35, 41). Our pooled studies included ≥ 35,230 residents, and 71% of them were in direct contact with COVID-19 patients. Female residents represented 53.2% of the participants, and young residents (R1, R2) represented 51% of the participants.

**Table 1:** Characteristics of the cross-sectional, anonymous online survey studies included in the systematic review.

**References/period survey**	**Country/setting**	**Specialty**	**Total eligible sample *N***	**Responders *N* (%)**	**Age mean (SD)/median (IQR)**	**Women *N* (%)**	**Year R *N* (%)**	**Direct-care C19(D)/Non-D C19 (ND) *N* (%)**	**Burnout tool**	**Newcastle Ottawa score**
**Whithout comparator group**										
Alkhamees et al. (36) (March 15th–April 23th, 2020)	Saudi Arabia National study	Psychiatry	150 R	121 (80.1%)	24–28 (67.8%) 29–33 (31.4%) 34–38 (0.8%)	51 (42.1%)	R1: 27 (22.3%) R2: 33 (27.3%) R3: 32 (26.4%) R4: 29 (24.0%)	NA	MBI-HSS	7
Chow et al. (23) (March 31th–April 6th, 2020)	USA National study	ORL	1,551 R	119 (7.9%)	NA	54 (45.4%)	R1: 26 (21.8%) R2: 24 (20.2%) R3: 20 (16.8%) R4: 26 (21.9%) R5: 23 (19.3%)	COVID-19 cases/100.000 people: Low: 20 = 42 Medium: 20–40 = 46 High: >40 = 31	SMDM OQB	6
Farsi et al. (37) (May, 2020)	Saudi Arabia National study	All specialties	346 R	328 (94.8%)	27.9 (2.25)	169 (51.5%)	R1: 81 (24.7%) R2: 76 (23.2%) R3: 76 (23.2%) R4: 71 (21.6%) R5: 24 (7.32%)	312 (95%) (D)	MBI-HSS	7
Kannampallil et al. (24) (April 10–25th, 2020)	USA Single center	All clinical	1,375 TR	393 (29%) T −261 (66.4%) R −132 (30.7%) F	NA NA NA	218 (55%) T NA NA	R1–R5 R1–R3 (80%)	218 (55%) (D) 175 (45%) (ND)	PFI	6
Khalafallah et al. (25) (May, 2 weeks, 2020)	USA National study	Neurosurgery	1,374 R	167 (12.2%) 111 complete response	< 30: 28 (25.2%) 30–40: 83 (74.8%)	57 (34.2%)	R1: 20 (18.0%) R2: 55 (49.5%) R3: 30 (27.0%) R4: 6 (5.4%)	102 (91.9%) (D) 9 (8.1%) (ND)	*a*MBI	6
Kaplan et al. (26) (April 14^th^-May 11th, 2020)	USA Single center	All specialties	991 TR	560 (56.6%)	< 35: 512 (91.4%) >35: 48 (8.6%)	280 (50.2%)	< R3: 207 (41.4%) >R3: 293 (58.6%)	560 (100%) (D)	Mini-Z	7
Mendoça et al. (42) (April, 2020)	Brazil All teaching hospital (São Paulo)	All specialties	1,392 R	Not calculate (convenience sample)	27.9 (3.0)	1,010 (72.5%)	R1: 493 (35.4%) R2: 407 (29.2%) R3: 273 (19.6%) R4: 153 (11%) R5: 53 (3.8%) R6: 13 (0.9%)	(69.8%) (D)	OLBI	2
Mion et al. (30) (March 7–21th, 2020)	France National study	Anesthesia (58%) Dermatology Others	1,055 R	NA	27 (2) (22–37)	609 (58%)	NA	100% (D)	MBI	5
Treluyer and Tourneux (31) (1st week of May, 2020)	France National study	Pedriatrics	1,300 R	340 (26.1%)	27 (25–28)	285 (83.8%) (79.5–87.6%)	R1: 79 (23.2%) R2: 74 (21.8%) R3: 81 (23.8%) R4: 96 (28.2%)	136 (40.0%) (D) 204 (60.0%) (ND)	MBI-HSS	6
Cravero et al. (44) (April 20th–May 11th, 2020)	International study^*^	All specialties^**^	1,420 TR 1,101 R 319 F	Not calculated (opportunistic sampling strategy)	≤ 25: 92 (30.9%) TR 26–30: 664 (59%) TR ≥31: 378 (33.4%) TR	767 (54%) TR NA NA	NA	623 (83%) R (D) 478 (75%) R (ND) 289 (53.7%) F (D) 158 (11%) F (ND)	*aa*MBI	5
Khooduruth et al. (39) (May 17th–June 16th, 2020)	Qatar National study	All specialties	640 T	127 (20%)	25–30: 94 (74%) 30–35: 31 (24%) >35: 2 (2%)	48 (37%)	R1–R2: 71 (56%) R3–R5: 57 (44%)	80 (63%) D 47 (27%) ND	ProQOL	6
**With comparator group**										
Aebischer et al. (32) (May 9th−14th)	Switzerland Single center	All specialties	227 R 550 S	Not calculated (snowball recruitment)	30 (28–32) 23 (21–24)	160 (70.5%) 412 (75%)	R1–R5	140 (61.7%) R (D) 51 (22.5%) R (ND) 160 (29%) S (D) 390 (71%) S (ND)	*aa*MBI	5
Al-Humadi et al. (45) (March 24th–May 15th, 2020)	USA Single center	All specialties	478 TR 901 P	113 (50.2%) TR 112 (49.8%) P	30.15 (2.76) TR 47.06 (3.01) P	58 (51.3%) TR 71 (63.4%) P	NA	NA	Two single items MBI	7
Civantos et al. (27) (April 14th−25th, 2020)	USA National study	ORL	1,614 R 2,849 P	165 (10.22%) R 184 (6.46%) P	26–30: 93 (56.4%) R 31–35: 66 (40.0%) R ≥36: 6 (3.6%) R 26–30: 1 (0.5%) P 31–35: 48 (26.1%) P ≥36: 135 (73.4%) P	76 (46.1%) R 61 (33.2%) P	NA	135 (82%) R (D) 25 (18%) R (ND) 125 (68%) P (D) 59 (32%) P (ND)	Mini-Z burnout assessment	6
Coleman et al. (28) (July, 2020)	USA National study	Surgery	10,991 R 16,257 P^*^	465 (4.2%) R 695 (4.3%) P^*^	26–30: 173 (37.6%) R 31–35: 241 (52%) R ≥36: 51 (11%) R 26–30: 10 (2%) P^*^ 31–35: 168 (24%) P^*^≥36: 508 (74%) P^*^	247 (53%) R 298 (43%) O NA	NA	381 (82%) R (D) 84 (18%) (ND) 473 (68%) P^*^ (D) 220 (32%) P^*^ (ND)	*a*MBI	6
Lasalvia et al. (33) (April 21th–May 6th, 2020)	Italy Single center	Medical	1,200 R 4,740 O 5,940 T	335 (27.9%) R 1,626 (34.3%) O 1,961 (33.01%) T	< 36: 633 (32.4%) T 36–55: 980 (50.1%) T >55: 343 (17.5%) T	1,471 (75%) T	NA	492 (25.5%) (D)¥	MBI-GS	7
Appiani et al. (43) (May, 2020)	Argentina Single center	All specialties	440 T	103 (34.1%) R 199 (65.9%) P^**^ 305 (69.38%) T	43.25 (12.0) T	48.7% T	NA	138 (45.7%) T	MBI	4
Elghazally et al. (38) (June–July, 2020)	Egypt Single center	All specialties	600 T	67 R 134 P 201 T (35.5%)	20–29: 89 (44.3%) T 30–39: 73 (36.3%) T >40: 39 (19.4%) T	131 (65.2%) T	NA	63 (31.3%) T	MBI	5
Bahadirli and Sagaltici (40) (July, 2020)	Turkey Istanbul University hospitals	All specialties in first line	629 emergency physicians	153 R 95 S 83 P 331 T (52.6%)	29 (27–35) T	142 (42.9%) T	NA	100%	MBI	5
**Before/during pandemic**										
Aziz et al. (29) (before July, 2020)	USA National study	General surgery	7,378 R approx. >year before	1,102 (14.6%)	NA	NA	R1: 20% R2–3: 41% R4–5: 38.1%	776 (70.4%) (D) 326 (29.6%) (ND)	Single question (MBI)	4
Degraeve et al. (34) (April 29th–May 3th, 2020)	Belgium National study	Urology	126 R Before/during	62 (49.2%)	25–27: 15 (24%) 29–30: 37 (60%) 31–35: 10 (16%) 29 (25–35) T	NA	R2: 15 (24.2%) R3: 15 (24.2%) R4: 16 (25.8%) R5: 5 (8.1%) R6: 6 (17.7%)	14 (22.5%) (D)	CBI, CBIPro, and CBIP subscales.	7
Osama et al. (41) (before July, 2020)	Pakistan Single center	Surgery	112 R Before/during	97 (86.6%)	30.50 (3.58)	45 (40.2%)	R1: 17 (15.2%) R2: 21 (18.8%) R3: 25 (22.3%) R4: 25 (22.3%) R5: 24 (21.4%)	NA	*d*MBI	6
Poelmann et al. (35) (December 30th–January 31th 2019; April 19th–May 5th, 2020)	Netherland National study	Surgery	317 R Before/during	317 B (81%) 313 D (72%)	32 (26–40) 32 (26–39)	47% 45%	R1–R6 R4: 68%	48.6% (D)	UBS	6
Σ residents	USA = 8 (35%) Nationals = 12 (52%)	All = 8 (35%) Surgery = 5 (22%)	*N* = ≥35,230^***^	*N* = 4,998 (34%)	51.4% (≤ 30 years)	53.2% women	51% (R1 + R2)	71% (D)	*N* = 15 MBI	6

W, Women; M, Men; EE, Emotional exhaustion; DP, Depersonalization; PA, Personal accomplishment; D, direct contact/COVID-19 patients; NDC, No direct contact; B, Burnout; O, Other health care professionals; F, Fellows; P, Physicians; P^*^, Early career physicians; P^**^, Emergency Physicians; R, Residents; S, Medical students; TR, Trainees (residents and fellows); T, Total participants; MBI, Maslash Burnout Inventory; aMBI, adapted MBI; aaMBI, Two single ítems from aMBI; dMBI, Dicotomizated MBI (yes/non); MBI-GS, MBI-General Survey; MBI-HSS, MBI-Health Survey; Mini-Z Burnout assessment; CBI, The Copenhagen Burnout Inventory; CBIP, CBI Personal dimension; CBIPro, CBI Professional dimension; CBIR, CBI Inventory personal dimension; OQB, One-question of Burnout; PFI, Stanford Professional Fulfillment Index; ProQOL, The Professional Quality of Life measure; SMDM, Shirom-Melamet Burnout Measure; UBS, Utrecht Burnout Scale.

^a^In the year before they were asked for burnout (aMBI).

^***^Three studies did not give the total number of residents.

¥All total sample, including residents and other healthcare.

Overall, 15 of 23 (65.2%) studies used the original MBI or its validated modifications as a method for burnout diagnosis or measurement (11, 12) (Table 1 and Supplementary Table 1). Fourteen studies (60.9%) provided the prevalence of burnout syndrome in residents (23–26, 29–31, 35–37, 39, 42–44), six studies (26.1%) provided the prevalence of high/low dimension (EE, DP, PA) (23, 30, 31, 33, 36, 37), and finally, three studies (13%) presented the results as mean (SD) or median (range) of each dimension (25, 30, 37). Some of the studies presented the burnout results in more than one way (Supplementary Table 2).

Eleven articles (47.8%) studied point-prevalence of burnout during the first wave of the pandemic without any comparator group (23–26, 30, 31, 36, 37, 42, 44) and eight studies (34.7%) compared the prevalence of burnout between residents and other physicians, students, or other health-workers during the period studied (27, 28, 32, 33, 38, 40, 43, 45). Four studies (17.4%) showed burnout prevalence in comparison with previously collected data (29, 34, 35, 41).

### 3.3 Risk of bias assessment

The quality assessment of the selected papers indicated that 3 of 23 studies (13%) presented a high risk of bias (29, 42, 43), and the rest of the studies had a low-to-moderate degree of bias (see Supplementary Table 3).

### 3.4 Meta-analysis results

#### 3.4.1 Overall prevalence of burnout

**Figure 2:**
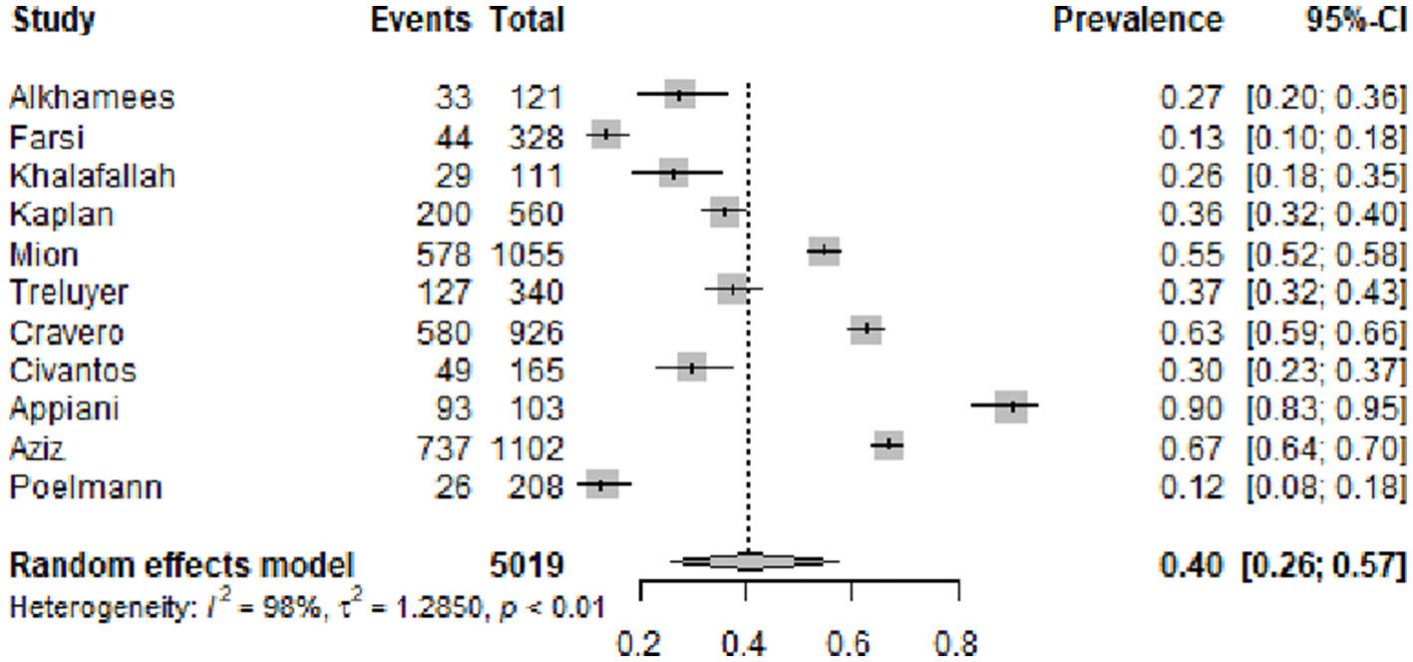
Estimated overall prevalence of burnout syndrome in residents during first wave of COVID-19 pandemic.

**Figure 3:**
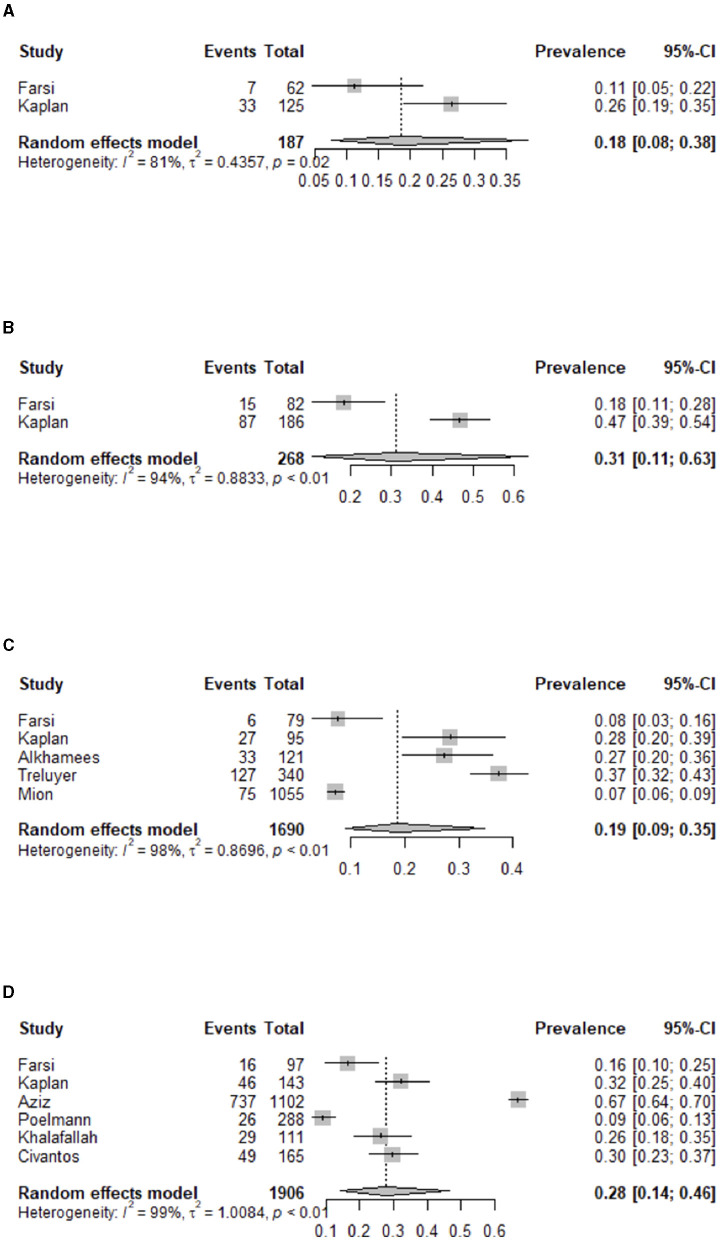
Estimated overall prevalence of burnout related to type of specialty **(A)** surgery, **(B)** internal medicine, **(C)** hospital based, and **(D)** others.

**Figure 4:**
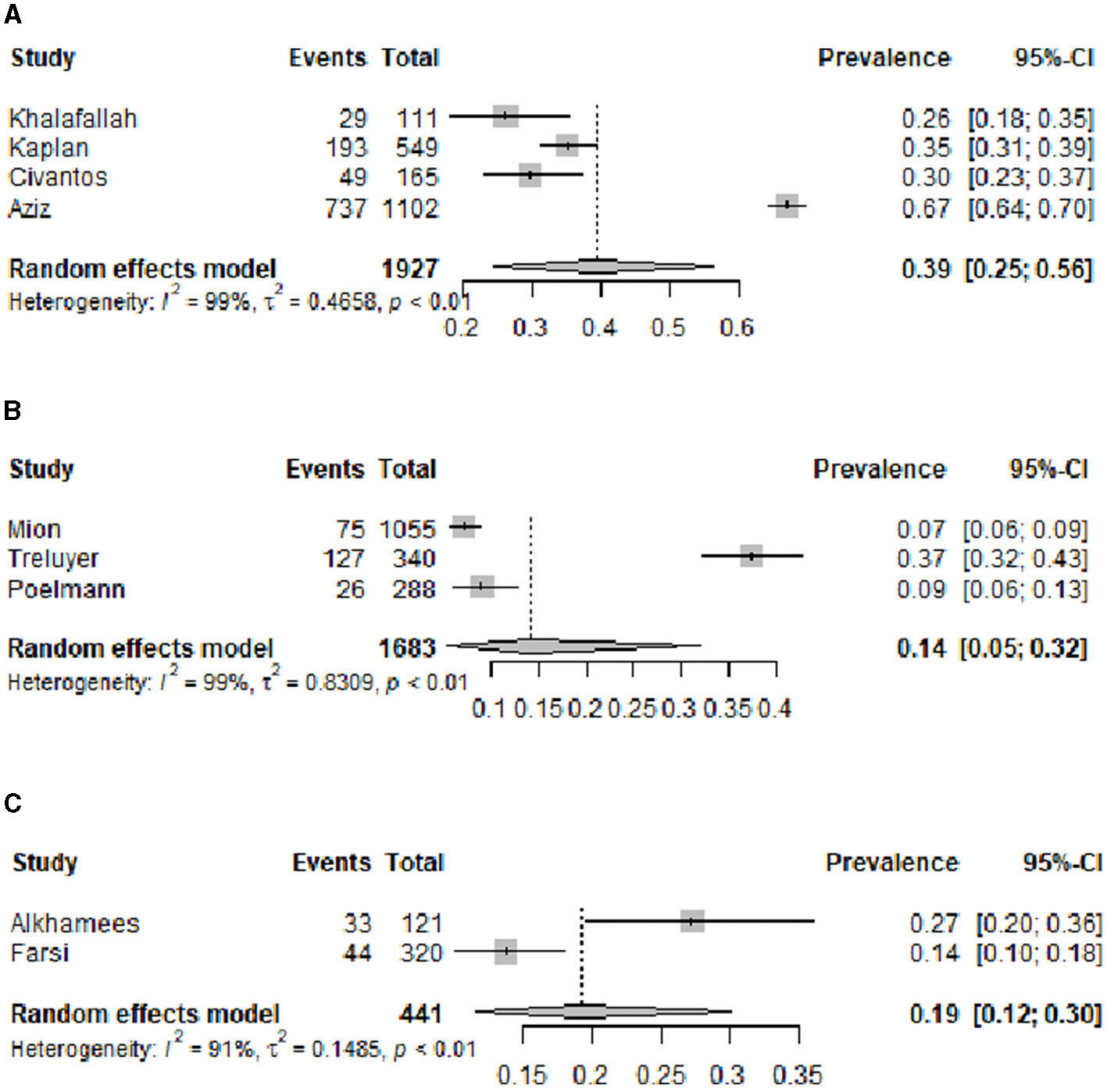
Estimated overall prevalence of burnout related geographical origin: **(A)** North-America, **(B)** Europe, and **(C)** Asia.

Figure 2 shows the funnel plot of the overall prevalence of burnout syndrome. From the 15 studies of burnout syndrome (categorical definition), we included, in the analysis, 11 studies that made the diagnosis of burnout syndrome with the original validated MBI (11, 12) or posterior validated versions (23, 25–27, 29, 30, 35–37, 43, 44). The estimated overall pooled prevalence (95% CI) was 0.40 (0.26–0.57). Figure 3 presents the funnel plot of the overall prevalence of burnout by type of specialty: surgical (general, neurosurgery, obstetrics, orthopedics, ophthalmology, urology, plastic surgery, thoracic surgery, and vascular surgery) (25–27, 29, 35, 37), with an overall prevalence (95% CI) of 0.27 (0.15–0.45); internal medicine and medical specialties (26, 37), with an overall prevalence (95% CI) of 0.31 (0.15–0.54); hospital-based specialties (anesthesia, radiology, and emergency) (26, 37), with an overall prevalence (95% CI) of 0.19 (0.10–0.32); and others (psychiatry, pediatrics, and dermatology) (26, 30, 31, 36, 37), with an overall prevalence (95% CI) of 0.18 (0.10–0.33). Figure 4 shows according to the geographic area where the study was conducted (grouped by continents), the overall prevalence (95% CI) of burnout in studies from North America (25–27, 29) was 0.39 (0.25–0.56), from Europe (30, 31, 35) was 0.14 (0.05–0.32), and from Asia (36, 37) was 0.19 (0.12–0.30).

#### 3.4.2 Overall prevalence of dimensions of burnout (high emotional exhaustion, high depersonalization, and low personal accomplishment)

**Figure 5:**
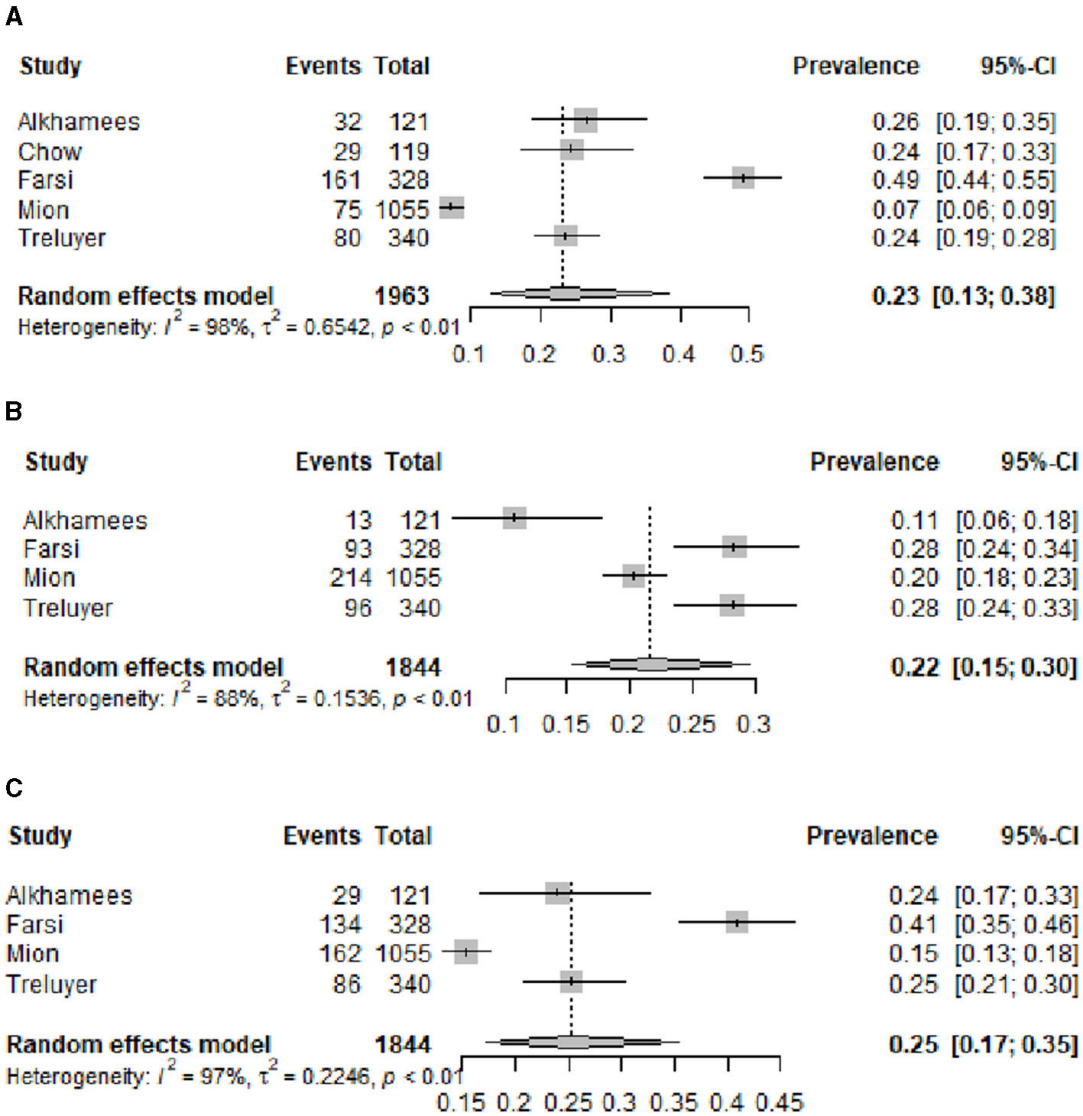
Estimated overall prevalence of burnout dimensions in residents during COVID-19 pandemic: **(A)** High emotional exhaustion, **(B)** high depersonalization, and **(C)** low personal accomplishment.

Five studies presented data on the percentage of high EE (23, 30, 31, 36, 37), and high DP (30, 31, 36, 37) and four studies of low PA (30, 31, 36, 37). The overall prevalence of high EE in residents according to MBI during the pandemic was 0.23 (0.13–0.38); the overall prevalence of high DP was 0.22 (0.15–0.30); and the overall prevalence of low PA was 0.25 (0.17–0.35). Figure 5 presents the three funnel plots, one for each dimension.

#### 3.4.3 Prevalence ratio of burnout in residents vs. non-residents

**Figure 6:**
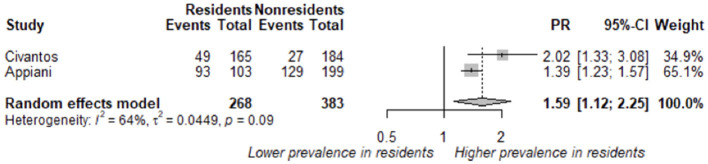
Estimated prevalence ratio (PR) of burnout comparing residents vs. others health care personal during COVID-19 pandemic.

In the analysis, we included two studies comparing the presence of burnout diagnosed using the original validated MBI tool or posterior validated versions in residents vs. non-resident healthcare workers (27, 43) (Figure 6). The estimated prevalence ratio of burnout associated with residency was 1.59 (1.12–2.25).

#### 3.4.4 Subgroup analysis: risk factors

Supplementary Figure 1 shows the funnel plots of the subgroup analysis-based studies that investigated sociodemographics and potential occupational risk factors for residents' burnout during the COVID-19 pandemic.

Five articles studied gender and civil status (26, 31, 36, 37, 44), three of them children in-charge (36, 37, 44), and four of them young/older residents (26, 31, 36, 37). All prevalence ratios of these sociodemographic variables were close to 1, and all the corresponding 95% confidence intervals included 1. The results of sociodemographic variables such as gender, civil status, or having children in-charge were very similar results between groups. In the included studies, the age of the residents was given in very different ways (interval, mean, median, and range), and it was difficult to synthesize quantitatively the results. We were able to analyze the residents by comparing young (R1-2) vs. old (R3-5) residents in some studies. However, although it seems that the younger residents were more likely to suffer burnout during the pandemic, the result of the meta-analysis was not statistically significant.

Occupational factors (daily exposure to COVID-19 patients, or >60 h per week working with COVID-19 patients) were studied in two (24, 37) and three (26, 30, 31) articles, respectively. None of the two meta-analyses revealed a PR that would have been statistically significantly different from others. Although 71% of residents were exposed to COVID-19 patients, most of the studies did not specify the frequency (days a week, hours a day, and first line) or the prevalence of burnout in those with or without direct contact with COVID-19 patients. Then, we could only use the data of a few studies in this analysis. Those residents who were highly exposed to COVID-19 patients seemed to be more likely to have burnout, but the results were not statistically significant.

**Figure 7:**
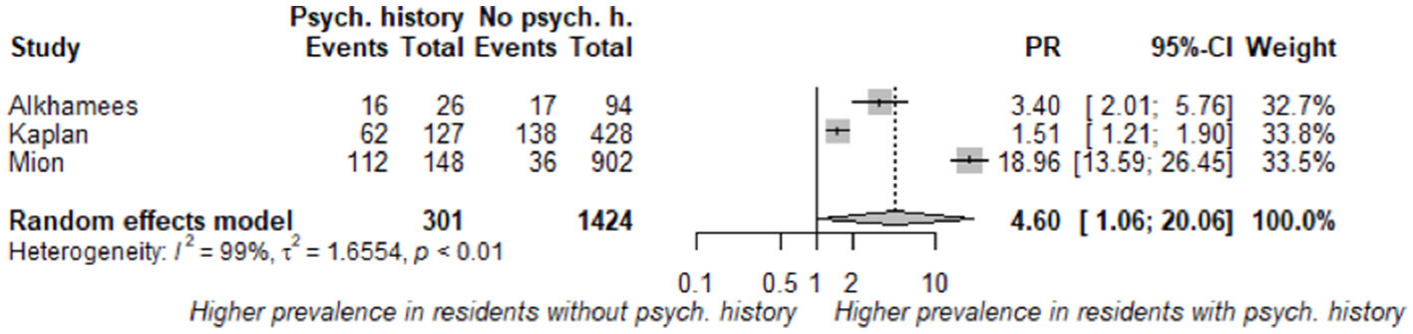
Estimated prevalence ratio (PR) of burnout associated with psychiatry history in residents during COVID-19 pandemic.

Figure 7 presents the funnel plot of the studies with data on psychiatric history (*N* = 3) (26, 30, 36). Using a random-effects model, the estimated PR was 4.60 (95% CI: 1.06–20.06). The prevalence of burnout during the COVID-19 pandemic was highly increased in those residents with a psychiatry history.

Supplementary Table 2 provides a description of the results of all articles included in the systematic review that analyzed risk factors associated with burnout in residents during a pandemic, some of which were not included in the meta-analysis for different reasons (different factors, burnout tools, design, or statistics).

#### 3.4.5 Publications bias results

Supplementary Figure 2 shows the funnel plot corresponding to the meta-analysis of the overall burnout prevalence. It does not show clear evidence of asymmetry and, hence, does not suggest publication bias. No funnel plot was drawn for the other meta-analyses because the number of studies included in these analyses was < 10 in all cases.

## 4 Discussion

The purpose of this systematic review was to synthesize and integrate the existing information related to the prevalence of burnout in residents and risk factors associated with the first wave of the COVID-19 pandemic. The systematic review included 23 observational studies involving 4,998 (34%) responders from 14 different countries, 71% of which were in direct contact with COVID-19 patients. A total of 87% of studies showed low-to-moderate risk of bias. We did not find publication bias. We could include 11 articles to study the pooled overall prevalence of burnout in residents during the first wave of the pandemic. The results of the funnel plot showed a pooled prevalence of 40% during a pandemic, with it being higher in North America (39%) than Europe (14%) or Asia (19%). In terms of specialties, residents of medical specialties (31%) were higher than surgical (27%) or other specialties (18%). Burnout in residents was more likely in those with a psychiatric history. We did not find any other sociodemographic or occupational risk factors associated with burnout in residents in this period.

### 4.1 Overall point prevalence

Surprisingly, *the overall point prevalence* of burnout during the COVID-19 pandemic found in the present review was similar to those figures reported in the systematic reviews before the pandemic (13, 14). Rodrigues et al.'s (13) systematic review with meta-analysis that included 26 cross-sectional studies from different countries, with ~5,000 residents of medical and surgical specialties evaluated with the MBI (10)^1^, found a global prevalence of 35.7% (95% CI: 26.8–43.5%). Low et al.'s (14) systematic review and meta-analysis included 61 cross-sectional and cohort observational studies, with more than 22,000 residents from different specialties and countries from Europe, Asia, and America, showing a global prevalence of burnout of 51.0% with the same tool (10)^1^ (95% CI: 45.0–57.0%). The results of our study were above the lower 95% CI of Lou's study and below the upper 95% CI of Rodrigues's study. Concerning the overall prevalence of MBI dimensions (EE, DP, and PA), in the review of Rodrigues et al. (13), 23 studies reported an overall prevalence rate of high EE of 38.9% (95% CI: 31.8–46.0%) and high DP of 43.6% (95% CI: 38.4–48.9%). The overall prevalence of low PA, studied in 20 articles, was 34.3% (95% CI: 21.3–47.2%). These figures would also be similar to those found in our study. Related to the overall prevalence of burnout in our study, medical specialties showed a little higher prevalence than surgical and other specialties, which is contrary to the results of Lou et al.'s (13) and Rodrigues et al.'s (12) studies before the pandemic period. During the COVID-19 pandemic, medical specialties were more on the frontline attending to COVID-19 patients. The overall prevalence in the North America continent was higher than in several European countries, which is in line with Low et al.'s study (13). However, we found less prevalence of burnout in Asian countries, opposite to Low et al.'s study, who found the highest overall prevalence in this continent. Our results may be explained due to the small number of Asian studies included.

From our review, the only study using the Copenhagen Burnout Inventory (CBI) (46) not included in the meta-analysis found a lower burnout prevalence during the pandemic than a previous assessment with the same tool and sample before the pandemic (34). Especially in personal exhaustion and professional exhaustion dimensions, associated to direct contact with COVID-19 patients.

Different factors could explain the similar burnout figures in residents found in our study during the first wave of the pandemic compared to pre-pandemic studies. The first factor may be indicative of a high level of resilience among residents during times of increased stress (34). But it can also be the effect of protective factors such as having more time to spend on reading/didactics, hobbies, or family/significant others due the reduction in usual clinical work or taking advantage of this period to carry out scientific work (28, 34). Either way, the pandemic situation has highlighted the importance of implementing wellness programs in institutions and their maintenance during times of health crisis (47, 48).

In our review, residents were more likely to burnout during the pandemic than other healthcare workers. However, we have to consider these data with great caution. We were able to make the comparison based only on the results of two studies. The studies included in the meta-analysis compared residents in training with other physicians (27, 43). Nevertheless, data from the literature show that other healthcare workers such as nursing professionals were one of the healthcare groups with the highest rates of burnout during the SARS-CoV-2 pandemic (49–51).

### 4.2 Potential risk factors

#### 4.2.1 Sociodemographic factors

##### 4.2.1.1 Gender

The number of physician women has increased in the last decades. Physician gender is a factor that has been proposed as a source of burnout, and some previous data suggested that women scored higher in the EE dimension than men, and men scored higher in DP and lower in PA dimensions (52). A recent systematic review of burnout and physician gender by Hoff and Lee (53) that included data from 45 studies from 2010 to 2019 showed that burnout is important both for female and male physicians, although women may experience it to a greater degree. Women had higher levels of EE dimension than men but authors did not find any evidence for gender differences in DP and PA dimensions (53). Some pieces of evidence suggest that the association between gender and burnout may vary among countries (54–56). In our review, 53.2% of the total residents were women and the differences were not statistically significant, which would imply that the pandemic affected the mental health of residents regardless of their gender.

##### 4.2.1.2 Age

It was not possible to analyze the association between age and burnout due to the lack of included studies reporting both variables in the same way. However, as the range of age of the included studies varied between 24 and 35 years old, the analyses of young (R1-2) and older (R3-5) residents could include age variables plus occupational factors. The early years of the residency period (R1-2) seem to be highly associated with burnout during the pandemic but again differences were not statistically significant. Both groups young (R1-2) and older (R3-5) have different patterns of needs and different associated stress factors. In the youngest residents, inexperience and the sudden change from student life to working life, and in the oldest, the feeling of having the skills of a specialist, mentoring the younger ones, and having to face the job search soon may be relevant (57). During the pandemic, it appears that all years of residence were similarly affected by the additional stress associated with it.

Prior to the pandemic, in addition to the year of residence, a review by Low et al. found that the older age of residents was significantly associated with a higher prevalence of burnout (14). On the opposite, Rodrigues et al. found that being young was a risk factor for burnout in residents (13). At present, it is not known whether the age of residents or other factors associated with age predispose them to burnout syndrome (58), so further research may be needed in this area.

##### 4.2.1.3 Civil status and care of children

Neither marital status nor having to care for children was a risk factor for resident physicians to present burnout during the pandemic in our study. Prior to the pandemic, being married or with a partner seemed to be a protective factor as well as the responsibility of caring for children against what could indicate an added stress factor (59). Parenting has a possible humanizing effect on residents, resulting in less detachment and depersonalization (60). In any case, these sociodemographic factors, as in our review, are not associated with the presence of burnout in most previous studies (61, 62).

#### 4.2.2 Psychiatric history

A 30-year seminar longitudinal study in the community showed that workers with a lifetime mood disorder, mainly of mood and anxiety disorders, have a higher risk for subsequent burnout (63). Although many studies indicate that psychiatric history and anxious-depressive disorders are high in medical residents, they have not always been found to be the risk factors for developing burnout during the residency (57, 64). The two pre-pandemic meta-analyses in residents did not study this risk factor (13, 14).

Residents with psychiatry history had a four-time higher risk of burnout during the pandemic peak stress in our meta-analysis. This increase in risk suggests that the subgroup of residents with a psychiatry history should be monitored closely during residency to prevent burnout development (9, 24).

#### 4.2.3 Occupational factors

The potential occupational risk factors measured in the different studies were very different from each other and difficult to synthesize the results (see Supplementary Figure 1).

Direct caring for COVID-19 patients was not a risk factor in our study for burnout in residents. In any case, the heterogeneity in the studies regarding the way this variable was assessed made it difficult to draw conclusions (direct contact vs. no-direct contact, number of hours/week, or COVID-19 patients attended…). In studies on other healthcare workers apart from residents (nurses, physicians,...), direct exposure to COVID-19 patients was a common risk factor for burnout (65–67).

The existence of clear protocols, the practical training regarding the protection measures, and the access and adequate use of PPE are all measures that ensure confidence and control, decreasing stress levels. Especially adequate PPE was demonstrated to protect against burnout in healthcare professionals during the pandemic (68). This review also points out these data (28, 44). However, each study assessed the topic in a different way.

Related to other occupational risk factors included in a single article, such as camaderie/support, increased work hours, interpersonal conflicts, or job satisfaction (26, 30, 33, 40), the data were not possible to be included in the meta-analysis.

### 4.3 Strengths and limitations

The strengths of this study were the inclusion of large samples of residents from national surveys, with a low-to-moderate risk of bias during the worldwide peak of the pandemic. However, our systematic review is not free of limitations. First, the most prevalent bias was the parameter of recruitment. Although most of the studies were nationals (including all possible candidates to participate), and none of the samples were random, some of the surveys used an opportunist sample or snowball recruitment. Second, there was a high variability between the response rates. Only five of the 23 included surveys had a satisfactory response rate (>60%). Web-based surveys have generally lower response rates than face-to-face or telephone interviews or mail surveys (69). Physicians as a professional group tend to present lower response rates than other collectives, and participation rates of 20% or less are not uncommon in web-based studies for physicians (70). Third, although the survey was anonymous in all cases, the participation was voluntary, meaning that it is possible that people more vulnerable were more likely to complete the survey, resulting in selection bias. Moreover, the cross-sectional design of studies made a causal relationship impossible, and as with all meta-analyses, there is always potential for publication bias as well as uncontrolled confounding variables. Finally, the results of the study cannot be generalized to all pandemic period as it refers only to the first wave frame. It would be very interesting to study the evolution of the overall prevalence and risk factors of burnout in residents during the complete COVID-19 pandemic period.

### 4.4 Conclusion

The prevalence of burnout in residents found in this systematic review and meta-analysis was similar to those obtained in the previous meta-analysis of burnout before the pandemic. Psychiatry history was associated with a higher risk of burnout in residents during the first wave of the COVID-19 pandemic, suggesting a high vulnerability of this subgroup of residents during the peak of the stress period.

## Data availability statement

The original contributions presented in the study are included in the article/Supplementary material, further inquiries can be directed to the corresponding author.

## Author contributions

RM-S: Conceptualization, Formal analysis, Methodology, Supervision, Writing – original draft. RN: Conceptualization, Data curation, Methodology, Writing – original draft, Writing – review & editing. VO: Conceptualization, Data curation, Methodology, Writing – original draft, Writing – review & editing. DH-M: Methodology, Validation, Writing – original draft, Writing – review & editing. KL: Formal analysis, Methodology, Supervision, Writing – review & editing. EV: Supervision, Writing – review & editing.

## Footnotes

^1^http://www.who.int/news/item/28-05-2019-burn-out-an-occupational-phenomenon-international-classification-of-diseases. Last enter: 27th April 2023.

## References

[1] Anmella G Fico G Roca A Gómez-Ramiro M Vázquez M Murru A . Unravelling potential severe psychiatric repercussions on healthcare professionals during the COVID-19 crisis. J Affect Disord. (2020) 273:422–44. 10.1016/j.jad.2020.05.061 32425275 PMC7228876

[2] Chen JA Chung WJ Young SK Tuttle MC Collins MB Darghouth SL . COVID-19 and telepsychiatry: early outpatient experiences and implications for the future. Gen Hosp Psychiatry. (2020) 66:89–95. 10.1016/j.genhosppsych.2020.07.002 32750604 PMC7347331

[3] Vieta E Pérez V Arango C. Psychiatry in the aftermath of COVID-19. Rev Psiquiatr Salud Ment. (2020) 13:105–10. 10.1016/j.rpsm.2020.04.004 38620300 PMC7177054

[4] Monninger M Pollok TM Aggensteiner PM Kaiser A Reinhard I Hermann A . Coping under stress. Prefrontal control predicts stress burden during the COVID-19 crisis. Eur Neuropsychopharmacol. (2023) 56:13–23. 10.1016/J.Euroneuro.2021.11.007 34894621 PMC8606266

[5] Manchia M Gathier AW Yapici-Eser H Schmidt MV de Quervain D van Amelsvoort T . The impact of the prolonged COVID-19 pandemic on stress resilience and mental health: a critical review across waves. Eur Neuropsychopharmacol. (2022) 55:22–83. 10.1016/j.euroneuro.2021.10.864 34818601 PMC8554139

[6] Wingenfeld K Schulz M Damkroeger A Rose M Driessen M. Elevated diurnal salivary cortisol in nurses is associated with burnout but not with vital exhaustion. Psychoneuroendocrinology. (2009) 34:1144–51. 10.1016/j.psyneuen.2009.02.015 19321266

[7] Chaukos D Chad-Friedman E Mehta DH Byerly L Celik A McCoy TH Jr . Risk and resilience factors associated with resident burnout. Acad Psychiatry. (2017) 41:189–94. 10.1007/s40596-016-0628-6 28028738

[8] Arnsten AFT Shanafelt T. Physician distress and burnout: the neurobiological perspective. Mayo Clin Proc. (2021) 96:763–69. 10.1016/j.mayocp.2020.12.027 33673923 PMC7944649

[9] Navinés R Olivé V Fonseca F Martin-Santos R. Work stress and resident burnout, before and during the COVID-19 pandemia: an up-date. Med Clin. (2021) 157:130–40. 10.1016/j.medcle.2021.04.005 35005240 PMC8721440

[10] ICD-11. Introduction. (2019). Available online at: https://www.icd.who.int/en (accessed April 27, 2023).

[11] Maslach C Jackson SE. The measurement of experienced burnout. J Organiz Behav. (1981) 2:99–113. 10.1002/job.4030020205

[12] Maslach C Jackon SE Leiter MP. Maslach Burnout Inventory Manual. 3rd ed. New York, NY: Ming Garden Editorial (2018).

[13] Rodrigues H Cobucci R Oliveira A Cabral JV Medeiros L Gurgel K . Burnout syndrome among medical residents: a systematic review. PLoS ONE. (2018) 13:e0206840. 10.1371/journal.pone.0206840 30418984 PMC6231624

[14] Low ZX Yeo KA Sharma VK Leung GK McIntyre RS Guerrero A . Prevalence of burnout in medical and surgical residents: a meta-analysis. Int J Environ Res Public Health. (2019) 16:e1479. 10.3390/ijerph16091479 31027333 PMC6539366

[15] Kemper KJ Schwartz A Wilson PM Mahan JD Schubert CJ Staples BB . Burnout in pediatric residents: three years of National survey data. Pediatric resident burnout-resilience study consortium. Pediatrics. (2020) 145:e20191030. 10.1542/peds.2019-1030 31843859

[16] Kocalevent RD Pinnschmidt H Nehls S Boczor S Siegert S Scherer M . Burnout and gratification crises in female and male physicians during postgraduate medical education in Germany. A longitudinal study. Psychother Psychosom Med Psychol. (2020) 70:319–29. 10.1055/a-1068-9843 31952095

[17] Dyrbye LN Burke SE Hardeman RR Herrin J Wittlin NM Yeazel M . Association of clinical speciality with symptoms of burnout and career choice regret among US resident physicians. J Am Med Assoc. (2018) 320:1114–30. 10.1001/jama.2018.12615 PMC6233627 30422299

[18] O'Connor P Lydon S O'Dea A Hehir L Offiah G Vellinga A . A longitudinal and multicentre study of burnout and error in Irish junior doctors. Postgrad Med J. (2017) 93:660–4. 10.1136/postgradmedj-2016-134626 28600343

[19] Cubero DI Fumis RR de Sá TH Dettino A Costa FO Van Eyll BM . Burnout in medical oncology fellows: a prospective multicenter cohort study in Brazilian institutions. J Cancer Educ. (2016) 31:582–87. 10.1007/s13187-015-0850-z 25952940

[20] Page MJ McKenzie JE Bossuyt PM Boutron I Hoffman TC Moher D. The PRISMA 2020 statement: updated guideline for reporting systematic review. Br Med J. (2021) 372:n71. 10.1136/bmj.n71 33782057 PMC8005924

[21] Wells GA Shea B O'Connell D Peterson J Wells V Losos M. The Newcastle-Ottawa Scale (NOS) for Assessing the Quality of Nonrandomized Studies in Meta-analyses. (2013). Available online at: http://www.ohri.ca/programs/clinical_epidemiology/oxford.asp

[22] Balduzzi S Rücker G Schwarzer G. How to perform a meta-analysis with R: a partial tutorial. Evid Based Ment Health. (2019) 22:153–60. 10.1136/ebmental-2019-300117 31563865 PMC10231495

[23] Chou DW Staltari G Mullen M Chang J Durr M. Otolaryngology resident wellness, training, and education in the early phase of the COVID-19 pandemic. Ann Otol Rhinol Laryngol. (2021) 3:1–11. 10.1177/0003-489420987194 33412923

[24] Kannampallil TG Goss CW Evanoff BA Strinckland JS McAlister RP Duncan J. Exposure to COVID-19 patients increases physician trainee stress and burnout. PLoS ONE. (2020) 15:e0237301. 10.1371/journal.pone.0237301 32760131 PMC7410237

[25] Khalafallah AM Lam S Gami A Dornbos DL Sivakumar W Johnson JN . A national survey on the impact of the COVID-19 pandemic upon burnout and career satisfaction among neurosurgery residents. J Clin Neurosci. (2020) 80:137–42. 10.1016/j.jocn.2020.08.012 33099336 PMC7438065

[26] Kaplan C Chi C Feingold J Kaye-Kauderer H Pietrzak RH Peccorolo L . Psychological consequences among residents and fellows during the COVID-19 pandemic in New York City: implications for targeted interventions. Acad Med. (2021) 96:1722–31. 10.1097/ACM.0000000000004362 34380941 PMC8603436

[27] Civantos AM Byrnes Y Chang C Prasad A Chorath K Poonia SK . Mental health among otolaryngology residents and attending physicians during the COVID-19 pandemic: National study. Head Neck. (2020) 42:1597–609. 10.1002/hed.26292 32496637 PMC7300862

[28] Coleman JR Abdelsattar JM Glocker RJ. COVID-19 pandemic and the lived experience of surgical residents, fellows, and early-career surgeons in the American College of Surgeons. J Am Coll Surg. (2021) 232:119–35e20. 10.1016/j.jamcollsurg.2020.09.026 33069850 PMC7561602

[29] Aziz H James T Remulla D Sher L Genyk Y Sullivan ME . Effect of COVID-19 on surgical training across the United States: a national survey of general surgery residents. J Surg Educ. (2021) 78:431–9. 10.1016/j.jsurg.2020.07.037 32798154 PMC7391955

[30] Mion G Hamann P Saleten M Plaud B Baillard C. Psychological impact of the COVID-19 pandemic and burnout severity in French residents: a national study. Eur J Psychiatry. (2021) 35:173–80. 10.1016/j.ejpsy.2021.03.005

[31] Treluyer L Torneux P. Burnout among paediatric residents during the COVID-19 outbreak in France. Eur J Pediatr. (2021) 180:627–33. 10.10007/s00431-020-03907-x 33410942 PMC7788161

[32] Aebischer O Weilenmann S Gachoud D Mean M Spiller T. Physical and psychological health of medical students involved in the COVID-19 response in Switzerland. Swiss Med Wkly. (2020) 150:w20418. 10.4414/esmw.2020.20418 33306812

[33] Lasalvia A Amaddeo F Porru S Carta A Tardivo S Bovo C . Levels of burnout among healthcare workers during the COVID-19 pandemic and their associated factors: a cross-sectional study in a tertiary hospital of a highly burdened area of north-east Italy. Br Med J Open. (2021) 11:e045127. 10.1136/bmjopen-2020-045127 33455940 PMC7813385

[34] Degraeve A Lejeune S Muilwijk T Poelaert F Piraprez M Svistakov I . When residents work less, they feel better: lessons learned from an unprecedent context of lockdown. Prog Urol. (2020) 30:1060–6. 10.1016/j.purol.2020.08.005 32917488 PMC7833413

[35] Poelmann FB Koeter T Steinkamp PJ Vriens MR Verhoeven B Kruijff S. The immediate impact of the coronavirus disease 2019 (COVID-19) pandemic on burn-out, work-engagement, and surgical training in the Netherlands. Surgery. (2021) 170:719–26. 10.1016/jsurg.2021.02.061 33820653 PMC7934663

[36] Alkhamees AA Assin H Alharbi HY Nasser A Alkhamees MA. Burnout and depression among psychiatry residents during COVID-19 pandemic. Hum Resour Health. (2021) 19:46. 10.1186/s12960-021-00584-1 33823857 PMC8022305

[37] Farsi A Alomar SA Kadi M Farsi S Algethamy H Reda B . Self-isolation during the COVID-19 pandemic is associated with increased risk of burnout among physician trainees: a cross sectional study. World Fam Med. (2021) 19:112–25. 10.5742/;EWFM.2021.93991

[38] Elghazally SA Alkarn AF Elkhayat H Ibrahim AK Elkhayat MR. Burnout impact of COVID-19 pandemic on healthcare professionals at Assiut University Hospitals, 2020. Int J Environ Res Public Health. (2021) 18:5368. 10.3390/ijerph18105368 34069955 PMC8157591

[39] Khoodoruth MAS Al-Nuaimi SK Al-Salihy Z Ghaffar A Khoodoruth WNC Ouanes S. Factors associated with mental health outcomes among medical residents exposed to COVID-19. BJPsych Open. (2021) 7:e52. 10.1192/bjo.2021.12 33583483 PMC8058844

[40] Bahadirli S Sagaltici E. Burnout, job satisfaction, and psychological symptoms among emergency physicians during COVID-19 outbreak: a cross-sectional study. Psychiatr Clin Psychopharmacol. (2021) 31:67–76. 10.5152/pcp.2021.20180 PMC11605308 39619354

[41] Osama M Zaheer F Saeed H Anees K Jawed Q Syed SH . Impact of COVID-19 on surgical residency programs in Pakistan; a residents' perspective. Do programs need formal restructuring to adjust with the “new normal”? A cross-sectional survey study. Int J Surg. (2020) 79:252–6. 10.1016/j.ijsu.2020.06.004 32526265 PMC7280820

[42] Mendoca VS Steil A Teixeira de Gois AF. COVID-19 pandemic in São Paulo: a quantitative study on clinical practice and mental health among medical residency specialties. São Paulo Med J. (2021) 139:489–95. 10.1590/1516-3180.2021.0109.R1.27042021 34287511 PMC9632538

[43] Appiani FJ Rodriguez Cairoli F Sarotto L Yaryour C Basile ME . Prevalence of stress, burnout syndrome, anxiety, and depression among physicians of a teaching hospital during the COVID-19 pandemic. Arch Argent Pediatr. (2021) 119:317–24. 10.5546/aap.2021.eng.317 34569739

[44] Cravero AL Kim NJ Feld LD Berry K Rabiee A Bazarbashi N . Impact of exposure to patients with COVID-19 on residents and fellows: an international survey of 1420 trainees. Postgrad Med J. (2020) 21:1–10. 10.1136/postgradmedj-2020-138789 33087533 PMC8543205

[45] Al-Humadi SM Caceda R Bronson B Paulus M Hong H Muhlrad S. Orthopaedic surgeon mental health during the COVID-19 pandemic. Ger Orthop Surg Rehab. (2021) 12:1–7. 10.1177/21514593211035230 34395046 PMC8361516

[46] Kristensen TS Borritz M Villadsen E Christensen KB. Copenhaguen Burnout Inventory: a new tool for assessment of burnout. Work Stress. (2005) 19:192–207. 10.1080/02678370500297720

[47] Loewenthal J Dyer NL Lipsyc-Sharf M Borden S Mehta DH Dusek JA . Evaluation of a yoga-based mind-body intervention for resident physicians: a randomized clinical trial. Glob Adv Health Med. (2021) 10:e21649561211001038. 10.1177/21649561211001038 33786209 PMC7961714

[48] Cotel A Golu F Pantea Stoain A Dimitriu M Socea B Cirstoveanu C . Predictors of burnout in healthcare workers during the COVID-19 pandemic. Healthcare. (2021) 9:304. 10.3390/healthcare9030304 33803286 PMC8001536

[49] Lima A Moreira MT Fernandes C Ferreira M Teixeira J Parola V . The burnout of nurses in intensive care units and the impact of the pandemic of SARS-CoV-2: protocol of a scoping review. Nurs Rep. (2022) 12:655–60. 10.3390/nursrep12030065 36135984 PMC9502256

[50] Xingyu D Zihong J Yiming X Zibei L Ziyang C Yixian Z . Psychological stress and coping strategies among frontline healthcare workers supporting patients with coronavirus disease 2019: a retrospective study and literature review. Ther Adv Respir Dis. (2022) 16:1–11. 10.1177/17534666221130215 PMC9742697 36476064

[51] Toscano F Tommasi F Giusino D. Burnout in intensive care nurses during the COVID-19 pandemic: a scoping review on its prevalence and risk and protective factors. Int J Environ Res Public Health. (2022) 19:12914. 10.3390/ijerph191912914 36232211 PMC9564773

[52] Houlkes I Winants Y Twellaar M Verdonk P. Development of burnout over time and the causal order of the three dimensions of burnout among male and female GPs. A three-wave panel study. BMC Public Health. (2011) 11:240. 10.1186/1471-2458-11-240 21501467 PMC3101180

[53] Hoff T Lee Do R. Burnout and physician gender. What do we know? Med care. (2021) 59:711–20. 10.1097/MLR.0000000000001584 34081678

[54] Dyrbye LN Shanafelt TD Balch CM Satele D Sloan J Freischlag J. Relationship between work–home conflicts and burnout among American surgeons: a comparison by sex. Arch Surg. (2011) 146:211–7. 10.1001/archsurg.2010.310 21339435

[55] Soler JK Yaman H Esteva M Dobbs F Asenova RS Katic M . Burnout in European family doctors: the EGPRN study. Fam Pract. (2008) 25:245–65. 10.1093/fampra/cmn038 18622012

[56] Kumar S. Burnout and doctors: prevalence, prevention and intervention. Healthcare. (2016) 4:37. 10.3390/healthcare4030037 27417625 PMC5041038

[57] Olivé V Navinés R Macías L López JA Ariz J Quesada S . Psychosocial and biological predictors of resident physician burnout. Gen Hosp Psychiatry. (2022) 78:68–71. 10.1016/j.genhosppsych.2022.07.007 35901627

[58] Amoafo E Hanbali N Patel A Singh P. What are the significant factors associated with burnout in doctors? Occup Med. (2015) 65:117–21. 10.1093/occmed/kqu144 25324485

[59] Martini S Arfken CL Churchill A. Burnout comparison among residents in different medical specialties. Acad Psychiatry. (2004) 28:240–2. 10.1176/appi.ap.28.3.240 15507560

[60] Collier VU McCue JD Markus A Smith L. Stress in medical residency: status quo after a decade of reform? Ann Intern Med. (2002) 136:384–90. 10.7326/0003-4819-136-5-200203050-00011 11874311

[61] Nituica C Bota OA Blebea J Cheng CI Slotman GJ. Factors influencing resilience and burnout among resident physicians—a National Survey. BMC Med Educ. (2021) 21:514. 10.1186/s12909-021-02950-y 34587948 PMC8479707

[62] Balendran B Bath MF Awopetu AI Kreckler SM. Burnout within UK surgical specialties: a systematic review. Ann R Coll Surg Engl. (2021) 103:464–70. 10.1308/rcsann.2020.7058 34192488 PMC10335046

[63] Rössler W Hengartner MP Ajdacic-Gross V Angst J. Predictors of burnout: results from a prospective community study. Eur Arch Psychiatry Clin Neurosci. (2015) 265:19–25. 10.1007/s00406-014-0512-x 24927954

[64] Ripp J Babyatsky M Fallar R Bazari H Bellini L Kapadia C . The incidence and predictors of job burnout in first-year internal medicine residents: a five-institution study. Acad Med. (2011) 86:1304–10. 10.1097/ACM.0b013e31822c1236 21869661

[65] Alonso J Vilagut G Mortier P Ferrer M Alayo I Aragón-Peña A . Mental health impact of the first wave of COVID-19 pandemic on Spanish healthcare workers: a large cross-sectional survey. Rev Psiquiatr Salud Ment. (2021) 14:90–105. 10.1016/j.rpsmen.2021.05.003 33309957 PMC7726524

[66] Slusarz R Cwiekala-Lewis K Wysokiński M Filipska-Blejder K Fidecki W Biercewicz M. Characteristics of occupational burnout among nurses of various specialties and in the time of the COVID-19 pandemic. Rev Int J Environ Res Public Health. (2022) 19:13775. 10.3390/ijerph192113775 36360655 PMC9657093

[67] Di Giuseppe M Nepa G Prout TA Albertini F Marcelli S Orrù G . Stress, burnout, and resilience among healthcare workers during the COVID-19 emergency: the role of defense mechanisms. Int J Environ Res Public Health. (2021) 18:5258. 10.3390/ijerph18105258 34069270 PMC8156145

[68] Morgantini LA Naha U Wang H Francavilla S Acar Ö Flores JM . Factors contributing to healtcare profesional burnout during the COVID-19 pandemic: a rapid turnaround global survey. PLoS ONE. (2020) 15:e0238217. 10.1371/journal.pone.0238217 32881887 PMC7470306

[69] Cunningham CT Quan H Hemmelgan B Noseworthy T Beck CA Dixon E . Exploring physician specialist response rates to web-based surveys. BMC Med Res Methodol. (2015) 15:32. 10.1186/s12874-015-0016-z 25888346 PMC4404667

[70] Dykema J Jones NR Piché T Stevenson J. Surveying clinicians by web: current issues in design and administration. Eval Health Prof. (2013) 36:352–81. 10.1177/016327871349663 23975760

