# Supplementary material for: Burnout in residents during the first wave of the COVID-19 pandemic: a systematic review and meta-analysis
Source: Front Psychiatry. 2024 Jan 24;14:1286101. doi: 10.3389/fpsyt.2023.1286101 (PMC10847582; doi:10.3389/fpsyt.2023.1286101)
Supplement: Supplementary file 1 [file Table_1.docx]

| Supplementary Table 1. Burnout questionnaires used in the included studies. | | |
| --- | --- | --- |
| **Burnout questionnaire** | **Items/ dimensions/scores** | **Burnout criteria** |
| **MBI**  Maslach Burnout Inventory (Maslach, Jackson, and Leiter, 1996).  *Mion et al., (2021),*^30^ *Appiani et al.,(2021),*^43^ *Elghazally et al.,(2021),*^38^ *Bahadirli and Sagaltici, (2021).*^40^ | 22 items and three dimensions:  -emotional exhaustion (MBI-EE, 9 items)  -depersonalization (MBI-DP, 5 items)  -personal accomplishment (MBI-PA, 8 items)  7-items Likert scale (0=never to 6=daily) | Burnout is defined when one /three criteria:  -High-EE (≥27 points), and/or  -High-DP (≥10), and/or  -Low-PA score (≤33) |
| **MBI-HSS**  Maslach Burnout Inventory-Health Survey  (Maslach and Jackson, 1981) (Dion and  Tessier, 1994).  *Treluyer and Tourneux, (2020).*^31^ | 22 items and three dimensions  -emotional exhaustion (MBI-EE, 9 items)  -depersonalization (MBI-DP, 5 items)  -personal accomplishment (MBI-PA, 8 items)  7-items Likert scale (0=never to 6=daily) | Burnout is defined when  -High-EE (≥30 points), and/or  -High-DP (≥12), regardless of Low-PA  -Low-PA score (≤33)  (Schaufeli et al., 2019; Dyrbye et al., 2009)  Valid tool in caregiver population |
| **MBI-HSS MP**  Maslach Burnout Inventory-Health Survey for Medical Personnel (Maslach et al., 2018).  *Alkhamees et al.,(2020),*^36^ *and Farsi et al.,(2020).*^37^ | 22 items and three dimensions  -emotional exhaustion (MBI-EE, 9 items)  -depersonalization (MBI-DP, 5 items)  -personal accomplishment (MBI-PA, 8 items)  and five profiles (P)  7-items Likert scale (0=never to 6=daily) | -High-EE, High-DP, Low-PA: Burnout P  -Low/Mod-EE, High-DP, Low/Mod-PA: Disengaged P  -High-EE,Low/Mod-DP,Low/Mod-PA: Overextended P  -Low/Mod-EE, Low/Mod-DP, Low-PA: Ineffective P  -Low-EE, Low-DP, and High-PA: Engaged P  (Leiter and Maslach, 2016) |
| ***d*MBI**  Maslach Burnout Inventory (dichotomized)  *Osama et al., (2020).*^41^ | 22 items, three dimensions:  -emotional exhaustion (MBI-EE)  -depersonalization (MBI-DP)  -personal accomplishment (MBI-PA)  Each item (yes=1 point and non=0 points) | Burnout is assessed as mean (SD) of total score.  (calculated by simple addition) and each  dimension) |
| **MBI-GS**  Maslach Burnout Inventory-General Survey  (Shaufeli et al., 1996).  *Lasalvia et al., (2021).*^33^ | 16 items and three subscales:  -emotional exhaustion (MBI-EE, 5-items)  -cynicism (CY(MBI-DP), 5-items)  -professional efficacy (MBI-PE, 6-items)  7-point Likert scale (0=never to 6=always) | Burnout is defined if  -High MBI-EE>2.20  -High MBI-CY>2 and  -Low MBI-PE<3.66  Valid tool for multiple cultural settings &occupations (Leiter and  Schaufeli, 1996; O´Connor et al., 2002; Rotenstein et al., 2018) |
| ***a*MBI**  Maslach Burnourt Inventory adapted-1.  *Coleman et al.,(2021),*^28^ *and Khalafallah et al.,*  *(2020).*^25^ | Nine-item questionnaire and 3 dimensions  -emotional exhaustion (MBI-EE, 3 items)  -depersonalization (MBI-DP, 3 items)  -personal accomplishment (MBI-PA, 3 items).  7-point Likert scale, and the total score (0-18) | Burnout is defined if  -High MBI-EE (≥13) or high MBI-DP (≥13)  Validated tool against MBI |
| ***aa*MBI**  Two single items derived from aMBI  (West et al., 2009).  *Aebisher et al.,(2020),*^32^ *Cravero et al.,(2020),*^44^  *Al-Humadi et al.,(2021),*^45^ *Aziz et al.,(2021).*^29^*** | Two-items and two dimensions:  -emotional exhaustion (MBI-EE)  -depersonalization (MBI-DP)  7-point Likert scale (0=never to 6=daily)  * One-single question from MBI | Burnout is defined if  -High MBI-EE (≥2) or  -High MBI-DP (≥2)  Validated tool against MBI (West et al., 2009;Dyrbye et al., 2014)  * |
| **Mini-Z Burnout assessment**  (Rohland et al., 2004).  *Civantos et al.,(2020),*^27^ *and Kaplan et al.,(2021)*.^26^ | A single-item measure  (range 1-5) | Burnout is defined as score ≥3  Validated tool against MBI. |
| **Self-reported burnout**  (Dolan et al., 2015).  *Chow et al., (2020).*^23^ | A single-item measure:*based on your definition of burnout, how would you rate your level of burnout*?  (1=No symptoms to 5=completely burned) | This item is dichotomized as ≤2 (no symptoms of  burnout) vs. ≥3 (1 or more symptoms)  Validated tool against MBI-EE (Dolan et al., 2015) |
| **SMDM**  Shirom-Melamet Burnout Measure  (Shirom and Melamed, 2006).  *Chow et al., (2020).*^23^ | 14 items and three dimensions:  -emotional exhaustion (EE)  -cognitive weariness (CW)  -physical fatigue (PF) | A SMDM index ≥4.40 suggest clinically relevant  burnout (Shilling et al., 2019) |
| **PFI**  Stanford Professional Fulfilment Index  (Tronkel et al., 2018).  *Kannampallil et al., (2020).*^24^ | A 16-items and three subscales:  -workload exhaustion (WE)  -depersonalization (DP)  -professional fulfilment (PF)  Score range for each subscales (0-4) | Burnout was determined from the average item  score of WE and DP scales). Scores≥1.33 was  considered burnout (Tronkel et al., 2018)  The burnout components of PFI (WE and DP)  correlate with those of MBI |
| **ProQOL**  The Professional Quality of Life measure  (Stamm BH, 2012).  *Khoodoruth et al., (2021).*^39^ | Three dimensions:  -professional satisfaction (PS)  -burnout (B)  -frightening experience at work (FEW) | The scores of the three categories:  -PS= <23 = poor level)  -Burnout = >41 higher risk score of burnout  -FEW= >43:higher risk score of FEW |
| **CBI**  The Copenhagen Burnout Inventory  (Kristensen et al., 2005).  *Degraeve et al.,(2020).*^34^ | 19 questions and three dimensions:  -personal exhaustion (CBIP)  -professional exhaustion (CBIPro)  -relationship exhaustion (CBIR) | The scores allow three categories:  -No worry (CBIP/R=0-13, CBIPro=0-15)  -Alertness (CBIP/R=14-17, CBIPro=16-19)  -Significant worrying (CBIP/R>17, CBIPro>19) |
| **OLBI**  The Odenburgh Burnout Inventory  (Demerouti & Bakker, 2008)  *Mendoca et al.,(2021).*^42^ | 16 items: 8 of exhaustion [2(R), 5, 8(R), 10, 12(R),14,16]; and 8 of disengagement [1, 3 (R), 6(R),7, 9(R), 11(R),13,15]. (R): reversed items  4-point Likert scale (1=strongly agree to  4=strongly disagree). | Low, medium, or high OLBI scores, based on  scores below 1 SD of the mean (M=2.15, SD=0.52)  -Low= ≤1.62  -Medium= 1.63-2.67  -High= ≥2.68 |
| **UBS**  Utrecht Burnout Scale (Schaufeli &  van Dierendoch, 2000)  *Poelmann et al.,(2021).*^35^ | 20 items and three subscales:  -emotional exhaustion (EE) (8 items)  -depersonalization (DP) (5 items)  -personal accomplishment (PA) (7 items)  7-point Likert scale (0=never to 6=daily) | Higher scores on EE and DP and lower PA  indicate burnout  Validated tool derived from MBI (Schaufeli &  van Dierendoch, 2000) |
