# Supplementary material for: Burnout in residents during the first wave of the COVID-19 pandemic: a systematic review and meta-analysis
Source: Front Psychiatry. 2024 Jan 24;14:1286101. doi: 10.3389/fpsyt.2023.1286101 (PMC10847582; doi:10.3389/fpsyt.2023.1286101)
Supplement: Supplementary file 2 [file Table_2.docx]

| Supplementary Table 2. Summary of results of prevalence of burnout and associated potential risk factors of the included studies. | | | | | | |
| --- | --- | --- | --- | --- | --- | --- |
| **Author/year/**  **period survey** | **Residents speciality**  **& N sample** | **Burnout**  **tool** | **Prevalence of burnout**  **N (%)** | | **Risk factors** | |
| ***Without comparator group*** | |  | **Burnout** | **No Burnout** | **Associated** | **Not associated** |
| Alkhamees et al., (2021)^36^  (March15^t^h –April 23^th^) | Psychiatry  -N=121 | MBI-HSS | 33 (27.3%) | 88 (72.7%) | -Depression (OR=8.88, 3.56-33.13; p=0.001)  -Mental help (OR=6.59, 2.60-16.70, p<0.05) | -Age, gender, civil status  -Raising children  -Year of R |
| Chow et al., (2020)^23^  (March 31^th^-April 6^th^) | OR  -N=119 | Single-item | 29 (24.4%)  (17.0%-31.8%) | 90 (75.6%)  (68.2%-83.0%) | -Women (p=0.001)  -R2 (p=0.007) | -Severity of Covid-19. |
| Farsi et al., (2020) ^37^  (May 2020) | All specialties  -N=328 | MBI-HSS | 44 (13.8%) | 276 (86.2%) | -Men (p=0.017), DC (p=0.05)  -Changed living place (p=0.035) | -Age, civil status  -Children  -Year of R |
| Kannampallil et al., (2020)^24^  (April 10^th^-25^th^) | All specialties  -N=393 | PFI | 160 (40.7%) | 233 (59.3%) | -DC (p=0.011). After adjusted by gender, civil status, children, year of R (p=0.0023) |  |
| Khalafallah et al., (2020)^25^ (May, two weeks) | Neurosurgery  -N=111 | *a*MBI | 29 (26.1%) | 82 (73.9%) | -No choose the speciality again (p=0.001)  -Lower post-graduate year (p=0.001)  -Alt. rotation (p=0.016) |  |
| Kaplan et al., (2021)^26^  (April 14^th^-May 11^th^) | All specialties  -N=560 | Mini-Z.  Single-item (EE) | **2**00 (35.8%) | 166 (29.7%) | -Speciality (AOR=2.8, 1.49-5.40; p=0.002)  -Psychiatry history (AOR=1.77, 1.07-2.91; p=0.03)  -Increased hours (AOR=2.40, 1.41-4.12; p**<**0.001)  -Other med.specialties (AOR=2.8, 1.49-5.40; p=0.002)  -Duty related worries (AOR=1.87, 1.33-2.67; p=0.001)  -Personal career (AOR=1.76, 1.20-2.59; p=0.004)  -Coping strategies: self-blame/venting (AOR=1.97, 1.27-3.05; p=0.002); substance use (AOR=2.34 (1.12-4.97; p=0.02), and not value by immediate supervisor (AOR=0.41, 0.21-0.79; p=0.008) | -Age, civil status  -COVID infection worries, infected others, -Resilience and camaderie |
| Mendoça et al., (2021)^42^  (April, 2020) | All specialties  -N=1392 | OLBI | 686 (49.2%) | 706 (50.7%) | -No clinical speciality (p<0.05). |  |
| Mion et al., (2021)^30^  (March 7-21^th^) | Anaesthesia (AR)  Dermatology  Others  -N=1055 | MBI | 578 (55%) | 472 (45%) | **-**N of calls (OR=1.12, 1.05-1.19; p=0.0004)  -Psychiatry history (OR=1.70, 1.23-2.34; p<0.001)  -Interpersonal conflict (OR=1.70, 1.23-2.34; p=0.002)  -Desire to quit speciality (OR=4.05,1.65-9.95; p=0.002)  -Fatigue (OR=1.12, 1.04-1.20; p=0.004)  -Job satisfaction (OR=0.89, 0.82-0.96; p=0.004)  -Depression (OR=1.34,1.01-1.80; p=0.046) | -Sleep  -Weekly hours  -Impact on personal life, being overwhelmed,  -COVID patients/week  -Feeling protected, clear procedures, being lonely, ethical conflicts  -Life satisfaction,  -Worried for loves one. |
| Treluyer & Tourneux  (2020)^31^  (1^st^ week of May) | Paedriatric  -N=340 | MBI-HSS | 127 (37.4%)  (32.2-42.7%) | 213 (76.5%)  (57.2-67.8%) | -Women (OR=0.47, 0.26-0.84; p=0.01)  - > 60h working (OR=3.98, 1.55-11.65; p<0.001)  -Anxiety score (OR=1.02, 1.01-1.03; p<0.001) | -Age, civil status  -Year of R  -N of shift, and DC. |
| Cravero et al., (2020)^44^  (April 20^th^-May 11^th^) | All specialties  N=926 | *aa*MBI | 580 (62%) | 345 (38%) | -Age (26-30: AOR=1.55, 1.16-2.40 to 36-40: 4.03, 2.12-7.63)  -Partnered (AOR=1.57, 1.06-2.33)  -Country: China (AOR=1.86,1.08-3.19); Saud Arabia (AOR=3.45,1.87-6.37); Taiwan (AOR=2.68,1.51-4.78)  -N COVID-19 patients [from 1-30 (AOR=1.90, 1.29-2.51) to >60 (AOR=4.03, 2.12-7.63)]  -PPE availability: compared to always, most of the time (AOR=1.99, 1.41-2.80) and sometimes AOR=2.81, 1.60-4.91)  -Colleague with COVID-19 (AOR=1.71, 1.26-2.37) | -Gender  -Children  -To be single or others  -Change of schedule |
| Khoodoruth et al., (2021)^39^ (May17^th^-June16^th^,2020) | All specialties  -N=127 R | ProQOL | N=0 (0%) | N=127 (100%) |  | -Gender  -Year of R. |
|  |  |  | **Burnout dimensions (High EE, High DP, Low PA)** | |  |  |
| Alkhamees et al., (2021)^36^  (March15^t^h –April 23^th^) | Psychiatry  -N=121 | MBI-HSS | High EE 32 (26.4%)  High DP 13 (10.7%)  Low PA 29 (24%) | Low EE 89 (74.6%)  Low DP 108 (89.3%)  High PA 92 (76%) | -Depression [AOR= 5.60 (1.94-16.12), p<0.05)]  -Depression [AOR= 3.33 (0.067-16.47), p<0.05)]  -Depression [AOR= 2.84 (1.02-7.94), p<0.05)] | -Age, sex, civil status, raising children, year R, previous need of mental help. |
| Farsi et al., (2020)^37^  (May 2020) | All specialties  -N=328 | MBI-HSS | High EE 161 (50%)  High DP 92 (28.8%)  Low PA 134 (41.9%) | Low EE 161 (50%)  Low DP 227 (71.2%)  High PA 186 (58.1%) | -Children (p=0.029), and daily DC (p=0.003)  -Men (p=0.05), and daily DC (p<0.001) | -Age, gender, civil status, year R  -Age, civil status, children, year R  -Age, gender, civil status, year R, and daily DC |
| Treluyer & Tourneux  (2020)^31^  (1^st^ week of May) | Paediatric  -N=340 | MBI-HSS | High EE 80 (23.5%)  High DP 96 (28.2%)  Low PA 86 (25.3%) | Low EE 260 (76.5%)  Low DP 244 (71.8%)  High PA 254 (74.7%) |  |  |
| ***With comparator group*** | |  | **Burnout N (%)**  **Residents Others** | |  |  |
| Civantos et al., (2020)^27^  (April 14^th^-25^th^) | ORL  -N=165 R  -N=184 O (staff physicians) | Mini-Z BA  Burnout Assessment | 49 (29.7%)  116 (70.3%) | 27 (14.7%)  157 (85.3%) | -B: R > O (OR: 0.28, 0.11-0.68; p=0.001)  -R+O was associated: with age (women), sex, surge status, and DC (p=0.005) |  |
| Appiani et al., (2021)^43^  (May 2020) | All specialties  -N=103 R  -N=199 O (heads and staff physicians) | MBI | 93 (90.3%) | 129 (63.7%) | -B: R > O (p<0.05)  -R+O was associated: DC, perception of non-adequate training, transient COVID-19 symptoms, hours of duty |  |
|  |  |  | **Burnout dimensions (EE, DP, PA)**  **N (%)** | |  |  |
| Al-Humadi et al., (2021)^45^  (March 24^th^-May 15^th^) | All specialties  -N=113 T  -N=12 (49.8%) O | Two single ítems of MBI | EE 18 (15.9%)  DP 23 (20.4%) | EE 25 (22.3%)  DP 13 (11.6%) | -EE and DP dimensions: R = O (p=0.736)-R+O was associated: negatively with age, and positively with history depression/anxiety and N of times on call in the last month |  |
| Coleman et al., (2021)^28^  (July) | Surgery  -N=465 R  -N=695 O (young surgeons) | *a*MBI | EE 257 (55%)  DP 180 (39%)  PA 209 (45%) | EE 385 (56%)  DP 204 (30%)  PA 307 (45%) | -EE dimension: R = O (p=0.96)  -DP dimension: R > O (p=0.002)  -PA dimension: R = O (p=0.94) |  |
|  |  |  | Burnout dimensions (HighEE, HighDP, LowPA) | |  |  |
| Coleman et al., (2021)^28^ (July) | Surgery  -N=465 R  -N=695 O (young surgeons) | *a*MBI | High B 209 (42%)  Low B 256 (38%) | High B 284 (58%)  Low B 411 (62%) | -High B: R = O (p=0.18) -R+O: High B (≥ 2 symptoms) was associated with sex (M), reduction elective operation, DC, perceived less adequate PPE access, and less support) (p<0.05) |  |
| Lasalvia et al., (2021)^33^  (April 21^th^-May 6^th^) | Medical specialties  -N=335 R  -N=1626 O (nurses, physicians, others healthcare staff, administratives) | MBI-GS | High EE 17 (34.9%)  Low EE 218 (65.1%)  High DP 112 (33.4%)  Low DP 223 (66.6%)  Low PA 244 (63.9%)  High PA 121 (36.1%) | High EE 752 (38.3%)  Low EE 584 (61.7%)  High DP 367 (22.6%)  Low DP 1259 (81.4%)  Low PA 911 (46.5%)  High PA 715 (43.5%) | -High EE: R < O [AOR: 1.82 (1.13-2.94) (p=0.014)] Sex, living conditions, DC length experience, job stress, psychological history, and interpersonal avoidance.-High DP: R > O [AOR: 2.02. (1.24-3.27) (p=0.004)] Psychological history, job stress, intern-personal avoid, and DC.-Low PA: R > O [AOR: 2.61 (1.71-3.98) (p=0.14)] Psychological history, interpersonal avoid |  |
|  |  |  | **Burnout dimensions [mean (SD) median (IQ)]** | |  |  |
| Aebischer et al., (2020)^32^  (May 9^th^-14^th^) | All specialties  -N=227 R  -N=550 S (N=296, students DC Covid-19/ N=254 NDC | *aa*MBI | N=140 frontline  EE 2 (1-4)  DP 2 (0-4) | N=160 frontline  EE 1 (0-3)  DP 1.5 (0-4) | **-**EE median dimension: R > O (p<0.01)  -DP median dimension: R = O (p=0.36) |  |
| Elghazally et al., (2021)^38^  (June-July 2020) | All specialties  -N=67 R  -N=134 O (assistant  lecture, specialist/lecturer, assist. professor/professors) | MBI | EE 27.4 (1.3)  DP 23.6 (1.3)  PA 26.1 (1.4) | EE 20.8 (1.7)  DP 14.5 (1.3)  PA 33.4 (1.6) | -EE mean dimension: R > O (p=0.01)  -DP mean dimension: R > O (p<0.001)  -PA mean dimension: R < O (p<0.001)  -R+O: EE (hours per day, age) (p<0.05), DP (hours per day, DC, mixed shits, age) (p<0.05) or PA (hours per day, age) (p<0.05) |  |
| Bahadirli and Sagaltici (2021)^40^  (July 2020) | Emergency physicians  -N=153 R  -N=95 S  -N=83 P | MBI | EE 23 (18-26)  R DP 10 (8-12)  PA 21 (18-25) | EE 19 (14-23)  S DP 8 (7-11)  PA 23 (18-27)  EE 22 (18-28)  P DP 10 (7-12)  PA 21 (16-25) | -EE mean dimension: R = P > S  -DP mean dimension: R = P, R > S, P > S  -PA mean dimension: R = S = P  **-**EE dimension: depression, stress, having Covid, 24h shifts, less job and career satisfaction  **-**DP: depression, 24h shifts, monthly income, less experience, and job satisfaction  **-**PA: job satisfaction |  |
| ***Before/During pandemic*** | |  | **Before During**  **[N (%)/mean (SD)]** | |  |  |
| Aziz et al., (2021)^29^  (before July) | General surgery  -N=1102 | One-question of burnout  (MBI) | 365 (33.1%) | 737 (66.9%) | -B: during > before pandemic.  -B > with year of R |  |
| Poelmann et al., (2021)^35^  (December 30^th^-January 31th) & (April 19^th^ –May 5^th^ 2020) | Surgery  -N=305 before  -N=288 during | UBS | 29 (9.5%) | 26 (9.0%) | -B: during = before pandemic  -Higher B in non-academic: before > during pandemic (p=0.007) | -Gender, children, fulltime /parttime, year of residency, Covid infection, or Covid/Non Covid ward |
| Degraeve et al., (2020)^34^  (April 29^th^-May 3rd) | Urology  -N=62 | CBI Mean (SD) | CBIP 7.26 (3.89)  CBIPro 9.02 (4.57) CBIR 4.42 (3.55) | CBIP 3.40 (3.38)  CBIPro 4.35 (4.56)  CBIR 3.03 (3.89) | -CBIP mean dimension: before > during pandemic [3.86 (2.61-5.09) (p<0.001)]  -CBIPro mean dimension: before > during pandemic **[**4.66 (3.26-6.06) (p<0.001)]  -CBIR mean dimension: before > during pandemic **[**1.38 (0.49-2.29) (p=0.003)]  -DC (CBIP and CBIPro) (p=0.003), and junior and senior residents (NS) |  |
| Osama et al., (2020)^41^  (before July) | Surgery specialties  -N=97 | *d*MBI | 14.75 (3.54) | 8.33 (2.34) | -B mean score: before > during pandemic (p<0.001)  -Working hours: during > before pandemic (p<0.001) |  |
|  | | | | | |  |

*Abbreviations:* B= Burnout; CBI=The Copenhagen Burnout Inventory, CBIP=CBI Personal dimension, CBIPro= CBI Professional dimension, and CBIR= CBI Inventory personal dimension; EE=Emotional exhaustion dimension; DC= direct contact with COVID-19 patients; DP= Depersonalization dimension; MBI=Maslach Burnout Inventory; *a*MBI= adapted MBI; *aa*MBI=Two single items derived from aMBI; *d*MBI= Dichotomized MBI (yes and non)]; MBI-GS= MBI-General Survey; MBI-HSS= MBI-Health Survey; Mini-Z Burnout assessment (range 1-5); NS= Not statistical significant; PFI=Stanford Professional Fulfilment Index; OQB=One-question of Burnout. SMDM= Shirom-Melamet Burnout Measure; M=Men. NDN=No direct contact with COVID-19 patients. O=Other health care professionals; PA= Personal accomplishment dimension; P= Physicians; R= Residents; R+O= total sample (residents plus others); TR= Trainees; S= Students; UBS=Utrecht Burnout Scal*e*; W=Women.

^a^Trainees (73% residents and 27%fellows).
