# Supplementary material for: Burnout in residents during the first wave of the COVID-19 pandemic: a systematic review and meta-analysis
Source: Front Psychiatry. 2024 Jan 24;14:1286101. doi: 10.3389/fpsyt.2023.1286101 (PMC10847582; doi:10.3389/fpsyt.2023.1286101)
Supplement: Supplementary file 3 [file Table_3.docx]

| Supplementary Table 3. Quality Assessment NOS | | | | | | | | | |
| --- | --- | --- | --- | --- | --- | --- | --- | --- | --- |
|  | **Selection (max. 4 starts)** | | | | **Comparatibility (max. 2 starts)** | | **Outcome**  **(max. 2 starts)** | | **NOS** |
| **Author/year** | **Represen**  **tativeness** | **Sample size** | **Non-responders** | **Ascertainment of the exposure**  **(absent/exclusion)** | **Control age/**  **gender** | **Control any other factor** | **Assessment** | **Statistical**  **test** | **Risk bias**  **score** |
| Alkhames et al., (2021)^36^ | ***** | ***** | ***** |  | ***** | ***** | ***** | ***** | **7** |
| Chow et al., (2020)^24^ | ***** | ***** |  |  | ***** | ***** | ***** | ***** | **6** |
| Farsi et al., (2020)^37^ | ***** | ***** | ***** |  | ***** | ***** | ***** | ***** | **7** |
| Kannampallil et al., (2020)^24^ | ***** | ***** |  |  | ***** | ***** | ***** | ***** | **6** |
| Khalafallah et al., (2020)^25^ | ***** | ***** |  |  | ***** | ***** | ***** | ***** | **6** |
| Kaplan et al., (2021)^26^ | ***** | ***** |  | ***** | ***** | ***** | ***** | ***** | **7** |
| Mendoca et al., (2021)^42^ |  |  |  |  |  | ***** | ***** |  | **2** |
| Mion et al., 2021^30^ | ***** |  |  |  | ***** | ***** | ***** | ***** | **5** |
| Treluyer & Tourneux, (2020)^31^ | ***** | ***** |  |  | ***** | ***** | ***** | ***** | **6** |
| Cravero et al., (2000)^44^ |  | ***** |  |  | ***** | ***** | ***** | ***** | **5** |
| Khooduruth et al., (2021)^39^ | ***** | ***** |  |  | ***** | ***** | ***** | ***** | **6** |
| Aebischer et al., (2020)^32^ |  | ***** |  |  | ***** | ***** | ***** | ***** | **5** |
| Al-Humadi et al., (2021)^45^ | ***** | ***** |  | ***** | ***** | ***** | ***** | ***** | **7** |
| Civantos et al., (2020)^27^ | ***** | ***** |  |  | ***** | ***** | ***** | ***** | **6** |
| Coleman et al., (2021)^28^ | ***** | ***** |  |  | ***** | ***** | ***** | ***** | **6** |
| Lasalvia et al., (2021)^33^ | ***** | ***** |  | ***** | ***** | ***** | ***** | ***** | **7** |
| Appiani et al., (2021)^43^ | ***** |  |  |  | ***** | ***** | ***** |  | **4** |
| Elghazally et al., (2021)^38^ | ***** | ***** |  |  | ***** | ***** | ***** |  | **5** |
| Bahadirli and Sagaltici, (2021)^40^ |  | ***** |  |  | ***** | ***** | ***** | ***** | **5** |
| Aziz et al., (2021)^29^ | ***** | ***** |  |  |  | ***** |  | ***** | **4** |
| Degraeve et al., (2020)^34^ | ***** | ***** |  | ***** | ***** | ***** | ***** | ***** | **7** |
| Osama et al., (2020)^41^ | ***** | ***** |  |  | ***** | ***** | ***** | ***** | **6** |
| Poelmann et al., (2021)^35^ | ***** | ***** | ***** | ***** |  | ***** | ***** |  | **6** |

The studies with more than 6 starts (maximum 8) were classified as low risk of bias, studies with 5 to 6 starts as moderate risk of bias, whilst studies with less than 5 starts were deemed as being of high risk of bias.
