# Supplementary material for: Burnout in residents during the first wave of the COVID-19 pandemic: a systematic review and meta-analysis
Source: Front Psychiatry. 2024 Jan 24;14:1286101. doi: 10.3389/fpsyt.2023.1286101 (PMC10847582; doi:10.3389/fpsyt.2023.1286101)
Supplement: Supplementary file 4 [file Data_Sheet_1.docx]

**Supplementary material (a).**

**Systematic review protocol** (The protocol was not registered)

**Review title**

Burnout in residents during the first-wave of COVID-19 pandemic: A systematic review and meta-analysis.

**Original language title**

English

**Stage of review at time of this submission**

**Started Completed**

Preliminary searches x x

Piloting of the study selection process x x

Formal screening of search results againts eligibility criteria x x

Data extraction x x

Risk of bias (quality) assessment x x

Data analysis x x

**Full name of the review team members**

-Ricard Navines. MD, PhD, Department of Psychiatry and Psychology, Hospital Clinic, University of Barcelona, IDIBAPS, CIBERSAM. Barcelona, Spain.

-Victoria Olive, MD, PhDfellowship, Department of Occupational Risk and Prevention, Hospital Clinic, and Department of Medicine, University of Barcelona. Barcelona, Spain.

-Diego Heredia, MD, PhD, Hospital Clinic, University of Barcelona, IDIBAPS, CIBERSAM. Barcelona, Spain.

-Klaus Langorh, PhD, Department of Statistics and Operations Research, Universitat Politècnica de Catalunya, Barcelona Tech, Barcelona, Spain.

-Eduard Vieta, MD, PhD, Hospital Clinic, University of Barcelona, IDIBAPS, CIBERSAM. Barcelona, Spain.

-Rocío Martin-Santos, MD, PhD, Hospital Clinic, University of Barcelona, IDIBAPS, CIBERSAM. Barcelona, Spain.

**Funding**

This systematic review has not received a specific grant form any funding agency, commercial or not-for profit sectors.

**Conflicts of interest**

EV has received grants and served as consultant, advisor or CME speaker for the following entities: AN.Biotics, AbbVie, Adamed, Angelini, Biogen, Biohaven, Boehringer-Ingelheim, Celon Pharma, Compass, Dainippon Sumitomo Pharma, Ethypharm, Ferrer, Gedeon, Ritcher, GH Research, Glaxo-Smith Kline, HMNC, Idorsia, Janssen, Lundbeck, Medincell, Merch, Novartis, Orion Corporation, Organon, Otsuka, Roche, Rovi, Sage, Sanofi-Avenis, Sunovion, Takeda, and Viatris, outside the present submmited protocol.

DHM has received CME-related honoraria and served as a consultant for Abbott, Angelini, Ethypharm Digital Therapy, and Jansen-Cilag, with no finncial or other relationships related to the subject of this paper.

The rest of authors declare that they have no known conflicts of interest.

**Others collaborators**

None

**Review question**

This is a systematic review of previously reported studies and will require no ethical approved focus on the study of:

1) Prevalence of Burnout in residents during the first wave of COVID-19 pandemic (March to July, 2020)

2) Associated risk factor to burnout in residents during the first wave of COVID-19 pandemic.

**Searches**

We will search the following electronic bibliographic databases: Web of Science, Scopus, Lilacs and PubMed database from 11^th^ of March 2020 until 1^st^ of October 2021.

**URL to search strategy**

The search strategy will be done using the following terms mixed with Boolean operators: “resident burnout” OR “trainee burnout” AND COVID-19.

**Condition or domain being studied**

Resident burnout syndrome during the first wave of COVID-19 pandemic

**Participants/population**

Studies that include medical residents in training during the COVID-19 pandemic

**Interventions, exposure**

Studies that assess the presence of burnout in residents during this period

**Comparator/controls**

We will compare residents with and without burnout during the first wave of pandemic period. Residents will be compared to other health workers (medical students, medical staff or others health workers) with and without burnout that have been assessed for burnout with the same tool during the same period of time (first wave of COVID-19 pandemic) and resident with burnout during and before pandemic.

**Types of study to be included**

According to the following predefined criteria: 1) Anonymous online cross-sectional survey studies, with and without comparator group and before-after studies. 2) Studies that have assessed burnout in residents during the pandemic with a validated burnout tool, i.e. Maslach Burnout Inventory. 3) Studies published in English or Spanish in a peer-reviewed journal.

The exclusion criteria would be 1) Other physician apart from residents in training; 2) Studies that assessed burnout exclusively outside the first wave of the pandemic period 3) Editorials, reviews, case report, commentaries, experimental, interventional and qualitative studies. 4) Studies with a N<50 participants.

**Context**

No restrictions of country, published in English or Spanish

**Primary outcome**

1) Overall prevalence of burnout syndrome during the COVID-19 pandemic or burnout dimensions (N and %; mean/SD/range).

**Timing and effect measures**

**Secondary outcomes**

1) Associated potential risk factor to burnout in residents during the first wave of COVID-19 pandemic (Prevalence ratios, PR)

**Data extraction**

Two independently authors will review all the studies (RN and VO). Differences in opinion will be resolved by consensus, and when necessary after discussion with a third researcher (RMS). A data schedule will be developed and use to retrieve information from the included studies.

A standardised, pre-piloted form will be used to extract data from the included studies for assessment of study quality and evidence synthesis. The variables to record for each study would be as follow: the author, year of publication, country, study design, sample size, gender, mean (SD) age and range, civil status, type of speciality, year of residence, direct contact with COVID-19 patients, burnout tool, burnout prevalence, burnout dimensions prevalence, risk/protector factors associated to burnout (sociodemographic, history of psychiatric disorder, year of residence; frequency or number of COVID-19 patients attended, positive COVID-19 one-self or in colleague; having adequate access to PPE, changes rotation/vacation; or increase/decrease weekly work hours. Two review authors will extract data independently; discrepancies will be identified and resolved through discussion (with a third author where necessary).

**Risk of bias (quality assessment)**

Two independently authors will review all the studies (DH and RMS). Differences in opinion will be resolved by consensus. The quality review of all studies would be performed using Newcastle Otawa Scale (NOS) for observational studies, with included eight items related to selection, comparison, and outcome. For each item a start is awarded, except for comparison that can receive up to two starts. The studies with more than 6 starts (maximum 9) were classified as low risk of bias, studies with 5 to 6 starts as moderate risk of bias, whilst studies with less than 5 starts were deemed as being of high risk of bias. In any doubt occurred, they were resolved by discussion.

**Strategy for data analysis**

First, we plan to provide a narrative systematic synthesis of the findings from the included studies around the burnout outcomes, and risk factors. Second, a quantitative synthesis will be used if the included studies are sufficiently homogeneous.

Where appropriate, we will carry out meta-analysis using, as a primary effect size, the proportion of burnout and dimensions [N (%) and 95%CI] or mean (SD), and secondary effect size the prevalence ratio of potential factors associated to burnout during Covid-19 pandemic [N (%) and 95%CI] or mean (SD).

Statistical heterogeneity among studies would be inspected through the I^2^ index (low heterogeneity ≤ 25%, moderate 50%, and high >75%) and the Cochrane´s Q statistic (p<1) and will be reported for all analyses. Independently of the corresponding x2 test for homogeneity, for the sake of coherence, random effects models will be employed for the estimation of all burnout prevalence and prevalence ratios. Furthermore, in the case of the estimation of the prevalence ratios the weights given to each study, i.e., the proportion of the total variability in the effect size estimates using random-effect models will be provided in the forest plots.

Subgroup meta-analysis estimates will be pooled based on population characteristics such as gender, civil status, children in charge, year of residency and specialization; burnout tool; and for different setting (direct or no direct contact with COVID-19 patients), if we found data to do it. A prevalence ratio (PR) of 1 means that the prevalence of the event, in this case burnout, is identical in the exposed and control or reference group whereas a PR greater (less) than 1 indicates that the prevalence of burnout is higher (lower) in the exposed group. The statistical significance at a 0.05 significance level of the estimated PR would be inferred from the 95% CI. If the CI will include the value 1, the estimated PR will not be statistically significant (p > 0.05) different from 1.

A graphical exploration of a potential publication bias by means of a funnel plot will be carried out if, at least, 10 or more studies are included in the analysis.

All analyses will be performed with the statistical software package R (The R Foundation for Statistical Computing), version 4.1.1; in particular we will use the contributed R package meta (Balduzzi et al., 2019).
