# Supplementary material for: Burnout in residents during the first wave of the COVID-19 pandemic: a systematic review and meta-analysis
Source: Front Psychiatry. 2024 Jan 24;14:1286101. doi: 10.3389/fpsyt.2023.1286101 (PMC10847582; doi:10.3389/fpsyt.2023.1286101)

Supplementary Figure 2. Funnel plot of included studies in the overall prevalence analysis to study the publication bias.

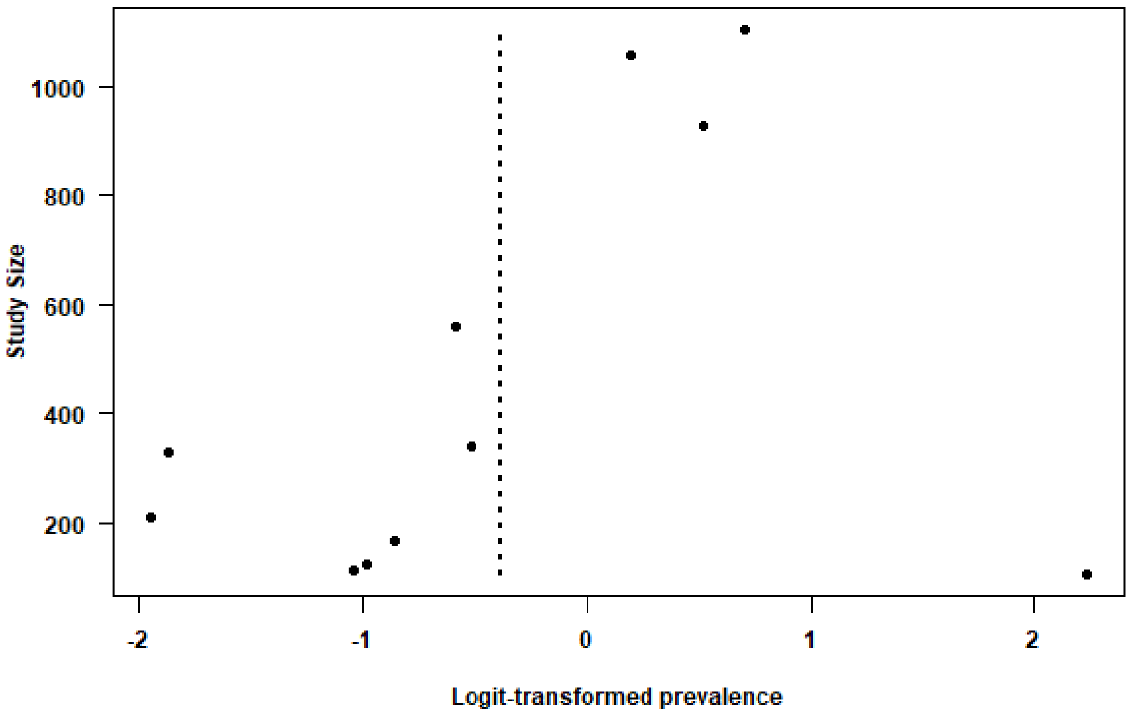

Supplement: Supplementary file 7 [file Image_2.pdf]
